# Transcription factor combinations that define human astrocyte identity encode significant variation of maturity and function

**DOI:** 10.1002/glia.24372

**Published:** 2023-04-08

**Authors:** Koby Baranes, Nataly Hastings, Saifur Rahman, Noah Poulin, Joana M. Tavares, Wei‐Li Kuan, Najeeb Syed, Meik Kunz, Kevin Blighe, T. Grant Belgard, Mark R. N. Kotter

**Affiliations:** ^1^ Department of Clinical Neurosciences University of Cambridge Cambridge CB2 0QQ UK; ^2^ Wellcome‐MRC Cambridge Stem Cell Institute, University of Cambridge Cambridge CB2 0AW UK; ^3^ The Bioinformatics CRO Sanford Florida 32771 USA

**Keywords:** astrocytes, human induced pluripotent stem cells, reprogramming, transcription factors, transplantation

## Abstract

Increasing evidence indicates that cellular identity can be reduced to the distinct gene regulatory networks controlled by transcription factors (TFs). However, redundancy exists in these states as different combinations of TFs can induce broadly similar cell types. We previously demonstrated that by overcoming gene silencing, it is possible to deterministically reprogram human pluripotent stem cells directly into cell types of various lineages. In the present study we leverage the consistency and precision of our approach to explore four different TF combinations encoding astrocyte identity, based on previously published reports. Analysis of the resulting induced astrocytes (iAs) demonstrated that all four cassettes generate cells with the typical morphology of in vitro astrocytes, which expressed astrocyte‐specific markers. The transcriptional profiles of all four iAs clustered tightly together and displayed similarities with mature human astrocytes, although maturity levels differed between cells. Importantly, we found that the TF cassettes induced iAs with distinct differences with regards to their cytokine response and calcium signaling. In vivo transplantation of selected iAs into immunocompromised rat brains demonstrated long term stability and integration. In conclusion, all four TF combinations were able to induce stable astrocyte‐like cells that were morphologically similar but showed subtle differences with respect to their transcriptome. These subtle differences translated into distinct differences with regards to cell function, that could be related to maturation state and/or regional identity of the resulting cells. This insight opens an opportunity to precision‐engineer cells to meet functional requirements, for example, in the context of therapeutic cell transplantation.

## Main Points

- Four transcription factor combinations were used to program human iPSCs into iAstrocytes and differences were investigated
- Fine‐tuning of the TF combination could produce iAs with greater therapeutic potential following transplantation

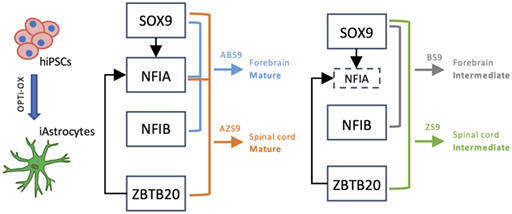

## 1 INTRODUCTION

Increasing evidence indicates that cellular identity can be reduced to the distinct gene regulatory networks (GRNs) active in a cell that are controlled by a distinct combination of transcription factors (TFs). Together, these form stable attractor states in Waddington's landscape (Waddington, 1957). This insight has resulted in reprogramming of cells based on forced expression of TFs (and other regulatory elements such as mammalian‐wide interspersed repeats). The expression of appropriate reprogramming factors induces conversion of cells from one lineage to another. A level of redundancy has been found, enabling different sets of TFs to induce broadly similar cell types (Ehrlich et al., 2017; Najm et al., 2013; Yang et al., 2013). In addition, varying the combination of TFs has been shown to result in different neuronal sub‐cell types (Black et al., 2020). An important question that has not yet been studied in detail is how the functional characteristics of cells induced by distinct sets of TFs relate to the cellular identity defined by the attractor state and whether those converge on a singular phenotype.

Astrocytes are an important brain cell type, contributing to synapse function (Kucukdereli et al., 2011; Perea et al., 2009; Pérez‐Alvarez & Araque, 2013; Stogsdill et al., 2017; Viola et al., 2009), myelination (Dutta et al., 2018; Lutz et al., 2009; Skripuletz et al., 2013), regulation of cerebral blood flow (Lind et al., 2013; Winship et al., 2007), blood brain barrier function (Abbott et al., 2006; W.‐L. Kuan et al., 2016), and immune regulation (Davalos et al., 2005; Esposito et al., 2006). Their dysfunction has also been implicated in multiple disease mechanisms (Booth et al., 2017; Molofsky et al., 2012; Phatnani & Maniatis, 2015; Verkhratsky et al., 2010). More recently, the transplantation of astrocytes has emerged as a promising therapeutic approach for neurological conditions, such as amyotrophic lateral sclerosis (ALS) (Izrael et al., 2018; Nicaise, 2015; Stoklund Dittlau et al., 2023), spinal cord injury (Davies et al., 2011) and Parkinson's disease (Hastings et al., 2022; Proschel et al., 2014; J.‐J. Song et al., 2017).

Human astrocytes are distinct from their rodent counterparts (Han et al., 2013; Oberheim et al., 2009; Zhang et al., 2016) and difficult to source. Traditional small‐molecule‐based stem cell differentiation can generate human astrocytes, however, it is limited by inconsistent and long protocols that are difficult to scale (Aldana et al., 2020; Izrael et al., 2018). A rapid and reliable method of generating astrocytes from human stem cells is therefore highly desirable. In addition, greater characterization of diverse protocols for induced human astrocyte generation could be of basic scientific and translational interest.

A number of TF combinations for reprogramming cells into astrocytes have been investigated. Expression of NFIA, NFIB, and SOX9 in combination, reprogrammed mouse embryonic and postnatal fibroblasts into astrocyte‐like cells (Caiazzo et al., 2015). In addition, successful reprogramming of human pluripotent stem cells (hPSCs) into induced astrocytes (iAs) was reported using NFIA or NFIA in conjunction with SOX9 (Li et al., 2018), or NFIB and SOX9 (Canals et al., 2018). The resulting iAs expressed canonical markers, supported calcium wave propagation and to enhanced neurite growth. Moreover, patterning of hPSCs with regional morphogens allowed for differentiation of iAs with specific regional phenotypes. Like other reprogramming paradigms, the diversity of combinations that can reprogram cells into astrocytes suggests that the responsible GRNs have a certain level of redundancy. Astrocyte identity may therefore be considered a poorly understood attractor state.

We previously demonstrated that in order to deterministically induce a new cell type from human induced pluripotent stem cells (hiPSCs), it is *necessary and sufficient* to ensure robust, transient expression of reprogramming factors (Pawlowski et al., 2017). Our work indicated that silencing of transgenes limits the efficiency of cellular reprogramming. We found that targeting an inducible TetOn system into genomic safe harbor sites enabled optimized transgene expression (OPTi‐OX) and, as a result, deterministic cell type conversion. This approach has been successfully applied for highly synchronized manufacturing of human neurons, skeletal myocytes, and oligodendrocytes (Pawlowski et al., 2017; Tourigny et al., 2019).

In the present study, we sought to take advantage of OPTi‐OX and investigate four different combinations of TFs that are able to convert hiPSCs into iAs. The aim was to shed light into the poorly understood GRN attractor state that defines astrocyte identity. We hypothesized that distinct TF combinations converge on a singular astrocyte attractor state with similar phenotype and function. Our data demonstrated that ‘astrocyte identity’ encompasses cellular states that are functionally highly distinct.

## 2 MATERIALS AND METHODS

### 2.1 Animals

All experiments on animals or involving the use of animal tissues were performed in accordance with a project license held under the Animals (Scientific Procedures) Act 1986, Amendment Regulations 2012, following ethical review by the University of Cambridge animal welfare and ethical review body. Neonatal Spraque Dawley rats and NIH‐Foxn1rnu immunocompromised nude rats were obtained from Charles River Laboratories.

### 2.2 Human iPSCs culture

HiPSCs (A1ATD‐iPSCs) (Yusa et al., 2011) were plated on Geltrex (Thermo Fisher Scientific) coated culture dishes and cultured in essential E8 medium (Life Technologies). Cells were passaged in small clumps using ReLeSR (StemCell Technologies) every 6–7 days.

#### 2.2.1 Gene targeting constructs and molecular cloning for hiPSCs

The pR26_CAG‐rtTA targeting vector was constructed as previously described (Bertero et al., 2016; Pawlowski et al., 2017). The four inducible AAVS1 targeting vectors were constructed by Gibson Assembly using Nebuilder HiFi DNA assembly cloning kit (New England Biolabs), in which either two or three inserts were ligated into the SpeI/EcoRI sites of our previously designed pAAV_TRE‐EGFP vector (Pawlowski et al., 2017): The pAAV_TRE‐ABS9 targeting vector was constructed by cloning the NFIA (Integrated DNA Technologies), NFIB (Integrated DNA Technologies) and SOX9 (Harvard Medical School) coding sequence, respectively. The pAAV_TRE‐BS9 targeting vector was constructed by cloning the NFIB and SOX9 coding sequence, respectively. The pAAV_TRE‐AZS9 targeting vector was constructed by cloning the NFIA, ZBTB20 (Integrated DNA Technologies) and SOX9 coding sequence, respectively. The pAAV_TRE‐ZS9 targeting vector was constructed by cloning the ZBTB20 and SOX9 coding sequence, respectively.

#### 2.2.2 Gene targeting of hiPSCs

Targeting of the human ROSA26 and AAVS1 loci was performed as described previously (Bertero et al., 2016; Pawlowski et al., 2017). Targeting of the hROSA26 locus was performed by nucleofection. Human iPSCs were dissociated into single cells with Accutase (Thermo Fisher Scientific), and 2 × 10^6^ cells were nucleofected (100 μL reaction volume; total of 12 μg of DNA, which was equally divided between the two gRNA/Cas9n plasmids and the targeting vector) using the P3 Primary Cell 4D‐Nucleofector kit (Lonza) and cycle CA‐137 of the Lonza 4D‐Nucleofector System. Nucleofected hiPSCs were plated onto Geltrex coated dish and cultured in essential E8 medium. Clone‐R (StemCell Technologies) was added for 48 h after nucleofection to promote cell survival. After 3–4 days, neomycin‐resistant hiPSCs were selected by adding G418 (50 μg/mL, Sigma‐Aldrich) for 7 days. Subsequently, individual clones were picked, expanded and finally analyzed by genotyping.

Targeting of the hAAVS1 locus, for the four TF combinations, was performed by nucleofection similar as for the hROSA26 locus (100 μL reaction volume; total of 12 μg of DNA, which was equally divided between the two AAVS1 ZFN plasmids and the targeting vector). After 3–4 days, resistant hiPSCs were selected by adding puromycin (1 μg/mL, Sigma‐Aldrich) for 4–5 days. Subsequently, individual clones were picked, expanded, and analyzed by genotyping.

Drug‐resistant hiPSC clones from targeting experiments were screened by genomic PCR to verify site‐specific transgene integration, to determine the number of targeted alleles, and to exclude off‐target integrations. PCRs were performed with LongAmp Taq DNA Polymerase (New England Biolabs). Sequencing confirmed the correct orientation of the cassette and the absence of mutations. See Table S1 for a full list of primer sequences. Clones that were homozygous for both the rtTA vector and the reprogramming cassette were chosen for further expansion and subsequent experiments.

### 2.3 Generation of the different cell types from hiPSCs

#### 2.3.1 Generation of human iAstrocytes

For iAs induction we followed Canals et al. protocol with some modifications (Canals et al., 2018). At a confluency of 70%–80%, targeted hiPSCs were dissociated with Accutase into single cells and plated onto Geltrex coated plates at a density of 50,000 cells per cm^2^. One day post plating, the medium was changed to essential E8 medium supplemented with 1 μg/mL Doxycycline (Dox; Sigma‐Aldrich) to initiate the induction. Then, on days 1 and 2, the medium was changed to FBS enriched medium: DMEM/F‐12 (Thermo Fisher Scientific), 10% FBS (Sigma‐Aldrich), 1% N2 supplement (Thermo Fisher Scientific), 1% Glutamax (Thermo Fisher Scientific), 1% Penicillin/Streptomycin (Thermo Fisher Scientific), 1 μg/mL Dox. For the following 3 days, the medium was gradually changed from FBS enriched medium to FGF enriched medium: Neurobasal (Thermo Fisher Scientific), 2% B27 (Thermo Fisher Scientific), 1% Non‐Essential Amino Acids (Thermo Fisher Scientific), 1% Glutamax, 1% FBS, 1% Penicillin/Streptomycin, 8 ng/mL FGF (Department of Biochemistry, University of Cambridge), 5 ng/mL CNTF (PeproTech), 10 ng/mL BMP4 (R&D Systems), 1 μg/mL Dox. On day 6, the medium was changed to full FGF medium. On day 7 post plating, cells were dissociated with Accutase and replated at same density on Matrigel coated plates or coverslips. One day after, medium was changed to FGF medium with 1 μg/mL Dox. From day 10 post plating onwards, half medium changes were made every other day with maturation medium: 1:1 DMEM/F‐12 and Neurobasal, 1% N2, 1% sodium pyruvate, 1% Glutamax, 5 ng/mL heparin‐binding EGF‐like growth factor (OriGene Technologies), 10 ng/mL CNTF, 10 ng/mL BMP4. Dox was withdrawn at day 14 post induction.

#### 2.3.2 Generation of human iNeurons

NGN2 targeted hiPSCs (Pawlowski et al., 2017; Tourigny et al., 2019) were dissociated into single cells with Accutase and plated onto Geltrex coated dishes at a density of 30,000 cells per cm^2^. Forward programming was initiated 24 h after the split. The induction was performed in DMEM/F‐12 supplemented with Glutamax (100×), Non‐Essential Amino Acids (100×), 50 μM 2‐Mercaptoethanol (Thermo Fisher Scientific), 1% Penicillin/Streptomycin, 1 μg/mL Dox. After 2 days of induction, the medium was switched to Neurobasal medium supplemented with Glutamax (100×), B27 (50×), 10 ng/mL BDNF (PeproTech), 10 ng/mL NT3 (R&D Systems), 1% Penicillin/Streptomycin, and 1 μg/mL Dox. Media changes were performed until D6 post induction, then half changes every other day. Dox was withdrawn at D6 post induction.

#### 2.3.3 Generation of neural precursor cells

NPCs were derived from human A1ATD‐iPSCs using small‐molecule‐based dual SMAD inhibition as previously described (Ehrlich et al., 2017).

### 2.4 Primary cultures

#### 2.4.1 Primary human astrocytes culture

Primary human astrocytes from the human brain (cerebral cortex) were purchased from ScienCell Research Laboratories at passage one. Cells were grown in astrocyte medium containing 2% FBS, astrocyte supplement and Penicillin/Streptomycin. All reagents were purchased from ScienCell Research Laboratories.

#### 2.4.2 Primary rodent cortical astrocytes culture

Primary mixed glial cultures were derived from the cerebral cortices of P0‐P2 neonatal Spraque Dawley rats (Charles River Laboratories) and were matured along the previous guidelines (McCarthy & De Vellis, 1980), with minor modifications (Syed et al., 2008). Mixed glia cells were maintained for 10 days in culture after which flasks were shaken for 1 h at 260 rpm on an orbital shaker to remove the loosely attached microglia, and then overnight at 260 rpm to dislodge oligodendrocyte precursors. Astrocyte cultures were maintained in glial culture medium (high‐glucose DMEM [Sigma‐Aldrich] supplemented with 10% FBS, glutamine [Sigma‐Aldrich] and 1% Penicillin/Streptomycin) for at least 2 weeks before passaging and platting for calcium imaging and multi‐electrode array (MEA) recording.

### 2.5 Immunocytochemistry

Cells on coverslips were fixed in 4% paraformaldehyde (diluted in PBS) for 15 min at room temperature and subsequently washed three times with PBS. The cells were then permeabilized with 0.1% Triton‐X‐100 (Sigma‐Aldrich) for 15 min at room temperature. Then, cells were blocked with 10% goat serum (Abcam) and 0.3% Triton X‐100 (diluted in PBS) for 30–45 min at room temperature. Subsequently, cells were incubated with appropriately diluted primary antibodies (Data S1) in 2% goat serum and 0.1% Triton X‐100 (diluted in PBS) at 4°C overnight. After three washes with PBS, the cells were incubated for 1 h at room temperature with corresponding goat fluorophore‐conjugated secondary antibodies (Alexa Fluor 488, 555, and/or 647; Invitrogen) in PBS supplemented with 1% goat serum. Nuclei were visualized with 4′,6‐diamidino‐2‐phenylindole (DAPI; Thermo Fisher Scientific). Cells were mounted on glass slides using Prolong mounting fluid (Invitrogen), dried overnight, and imaged using a Zeiss LSM 710 confocal microscope (Leica).

### 2.6 Immunohistochemistry

Fixed free‐floating brain slices were incubated with 0.5% TritonX‐100 and 5% goat or donkey serum in PBS overnight to permeabilize the tissue and block non‐specific antibody binding, then incubated in PBS, 0.3% TritonX‐100 and 3% serum for 2 days with appropriately diluted primary antibodies (Data S1). After washes in PBS, the staining was visualized either by immunofluorescence as described in Section 2.5 or diaminobenzidine (DAB). Staining omitting primary antibodies was performed in all immunohistochemical experiments to serve as negative controls. Slices were mounted on glass slides using FluorSave (Millipore) mounting fluid, dried overnight, and imaged using Zeiss 710 confocal microscope for immunofluorescence or Olympus C.A.S.T. grid stereological system for cell counting based on DAB staining.

### 2.7 Morphological analysis

IHC images were analyzed and processed in ImageJ software. Astrocytic process length from brain slices was measured using “Simple Neurite Tracer” ImageJ plugin by manually selecting 30 processes per condition of either iAstrocytes (transplanted into the left hemisphere), primary human astrocytes (transplanted into the right hemisphere), both panGFAP—human GFAP co‐labeled, or host rodent astrocytes from areas where no human GFAP signal could be detected.

### 2.8 Reverse transcription quantitative PCR

Total RNA was extracted from iAs at day 14 post induction for each line using GenElute mammalian total RNA miniprep kit (Sigma‐Aldrich) according to the manufacturer's protocol. cDNA synthesis was performed with the Maxima First Strand cDNA Synthesis Kit (Thermo Fisher Scientific). Applied Biosystems SYBR Green PCR Master Mix was used for qPCR. Samples were run on the QuantStudio 6 Flex Real‐Time PCR System machine. All samples were analyzed in technical duplicates and normalized to the house‐keeping gene Porphobilinogen Deaminase 1 (*PBGD1*). Results were analyzed with the ΔΔCt method. See Table S2 for a full list of primer sequences.

### 2.9 RNA sequencing analysis

#### 2.9.1 RNA from cell cultures

Total RNA was extracted from hiPSCs and from all four iAs lines using GenElute mammalian total RNA miniprep kit according to the manufacturer's protocol. RNA quality and quantity were evaluated using Qubit 4 fluorometer (Invitrogen) with the Qubit RNA BR assay kit (Invitrogen). RNA sequencing libraries were constructed by Novogene UK.

#### 2.9.2 RNA from fixed tissues

Astrocyte graft‐enriched tissue was manually dissected from rat striata in brain slices using the needle track and human GFAP staining as a visual guide. Total RNA was subsequently extracted from the graft‐enriched tissue using RNeasy Plus Micro Kit (Qiagen) according to the manufacturer's protocol. RNA quality and quantity were evaluated by bioanalyzer. RNA sequencing libraries were constructed using Takara Pico v3 kit by the NGS library facility, University of Cambridge, United Kingdom.

A detailed description of the analysis can be found in the Data S1.

### 2.10 Cytokine stimulation

At 14 days post induction, iAs were incubated for 8 h with or without 10 ng/mL IL‐1β (PeproTech) in fresh maturation medium. After stimulation, RNA isolation and qPCR were performed as described above (Section 2.8).

### 2.11 Calcium imaging

Intracellular Ca^2+^ levels were measured with radiometric dye fura‐2 largely in accordance with a previously published report (Rahman & Rahman, 2017). Briefly, rat astrocytes, iAs, iPSCs, and NPCs cultured on 35‐mm glass‐bottom Petri dishes (Ibidi), were loaded with 5 μM fura‐2/AM (eBioscience) for 40 min at 37°C, 5% CO_2_ in culture medium. Cells were then washed and kept in pre‐warmed calcium‐free HBSS buffer for 30 min. Ratio fluorescence images were captured using a QIClick™ digital CCD camera (QImaging) mounted on a Nikon Eclipse Ti‐S Microscope. Consecutive excitation was provided by a Dual OptoLED Power Supply (Cairn), alternating between both 355 nm (F 355) and 380 nm (F 380) wavelength LEDs. Emission fluorescence was collected at 510 nm (470–550 nm). After 60s baseline, 100 μM adenosine triphosphate (ATP; Sigma‐Aldrich) was added to the bath. 12‐bit images were acquired every 3 sec with MetaFluor® Fluorescence Ratio Imaging Software (Molecular Devices, United States). The fluorescence intensity ratio (F 355/F 380) was extracted, corrected for autofluorescence, and normalized to baseline to calculate intracellular Ca^2+^ levels. All imaging experiments were performed at room temperature.

### 2.12 MEA recordings

At day 3 post induction, human iNeurons (iNs) were dissociated into single cells and replated on PDL/Geltrex coated wells of CytoView MEA 48 (Axion Biosystems) together with each of the four iAs lines or with rat cortical astrocytes, at a ratio of 1:1. iNs monocultures were plated as a control. Co‐cultures were then maintained in 1:1 iNs medium:iAs maturation medium for the duration of recordings. Recordings were performed for 20 min every week until day 42 post induction of iNs using Maestro Pro MEA system (Axion Biosystems).

### 2.13 Animal surgery and cell transplantation

Transplantation of iAs and primary human astrocytes was conducted using NIH‐*Foxn1rnu* immunocompromised nude rats (Charles River Laboratories, *n* = 6), to minimize rejection of the xenograft with the aim of assessing the ability of human astrocytes to survive, differentiate, and integrate into the host brain. Three hundred thousand cells per 4 mL of sterile DMEM/DNAse medium per hemisphere were intracerebrally injected into the striatum under general anesthesia. Infusion was done over a 4‐min period using a 10 μL Hamilton syringe targeted at stereotaxic coordinates (relative to bregma and the dural surface): −4.4 mm anterior (*A*), −1.0 mm lateral (*L*) and −7.5 mm vertical (*V*) with the incisor bar set 2.3 mm below the interaural line. The syringe needle was kept in place for 2 min after the infusion to allow to minimize the efflux of the cell graft (W. L. Kuan et al., 2007). Animals were then culled by transcardial perfusion with 4% paraformaldehyde followed by decapitation at regular timepoints (2 rats after 2 weeks, 4 rats after 3 months). All procedures were conducted in accordance with the University of Cambridge ethics and animal care standards. Brains were removed and immersed in 30% sucrose in PBS for 2 days, and then sliced into 35 mm thick free‐floating sections for subsequent immunohistochemistry (IHC) analysis as described above (Section 2.7).

#### 2.13.1 Stereology

The total number of human nuclear antigen‐positive cells in the rat striatum at 2 weeks post‐transplantation was quantified using a modified 2D stereological sampling protocol (W. L. Kuan et al., 2019) with the Olympus C.A.S.T. grid stereological system. The total graft area was estimated using one‐sixth of the total sections, from which sampling regions were selected randomly. Graft areas were defined by the dense presence of human nuclei as no significant migration was observed at that time‐point.

### 2.14 Statistical analysis

Data are presented as the mean ± SD unless otherwise stated. For quantification of experimental results and determination of statistically significant differences, data were analyzed in GraphPad Prism software. For comparisons between two groups, the unpaired student *t* test was used. For multiple comparisons, the one‐way analysis of variance (ANOVA) was used with Dunnett's or Tukey's multiple comparisons test.

## 3 RESULTS

### 3.1 Selection of candidate TF combinations for iAstrocyte reprogramming

Candidate TFs for reprogramming hiPSCs into an astrocytic phenotype were selected based on previous reports of reprogramming protocols used for iAs, and on TFs that play key roles during astrocyte specification during development which have been tested in our lab. Previous reprogramming protocols involved forced expression of NFIA, NFIB and SOX9 in various starting cell types and combinations: NFIA, NFIA+SOX9 (Li et al., 2018) and NFIB+SOX9, NFIA+NFIB+SOX9 (Canals et al., 2018) in hPSCs, and NFIA+NFIB+SOX9 in mouse fibroblasts (Caiazzo et al., 2015). In addition, we included ZBTB20, a TF that has not yet been investigated in the context of astrocyte reprogramming. During development, Zbtb20 interacts with NFIA and Sox9 to promote astrocytogenesis (Nagao et al., 2016). Moreover, preliminary experiments suggested that ZBTB20 can be used to induce astrocyte identity, suggesting a certain level of redundancy with regards to TF programs. The present study therefore investigated the following four TFs combinations with regards to their ability to reprogram hiPSCs into an astrocyte identity: NFIA‐NFIB‐SOX9 (ABS9); NFIB‐SOX9 (BS9); NFIA‐ZBTB20‐SOX9 (AZS9), and ZBTB20‐SOX9 (ZS9).

### 3.2 Forced expression of all four TF combinations successfully downregulated pluripotency markers

**FIGURE 1:**
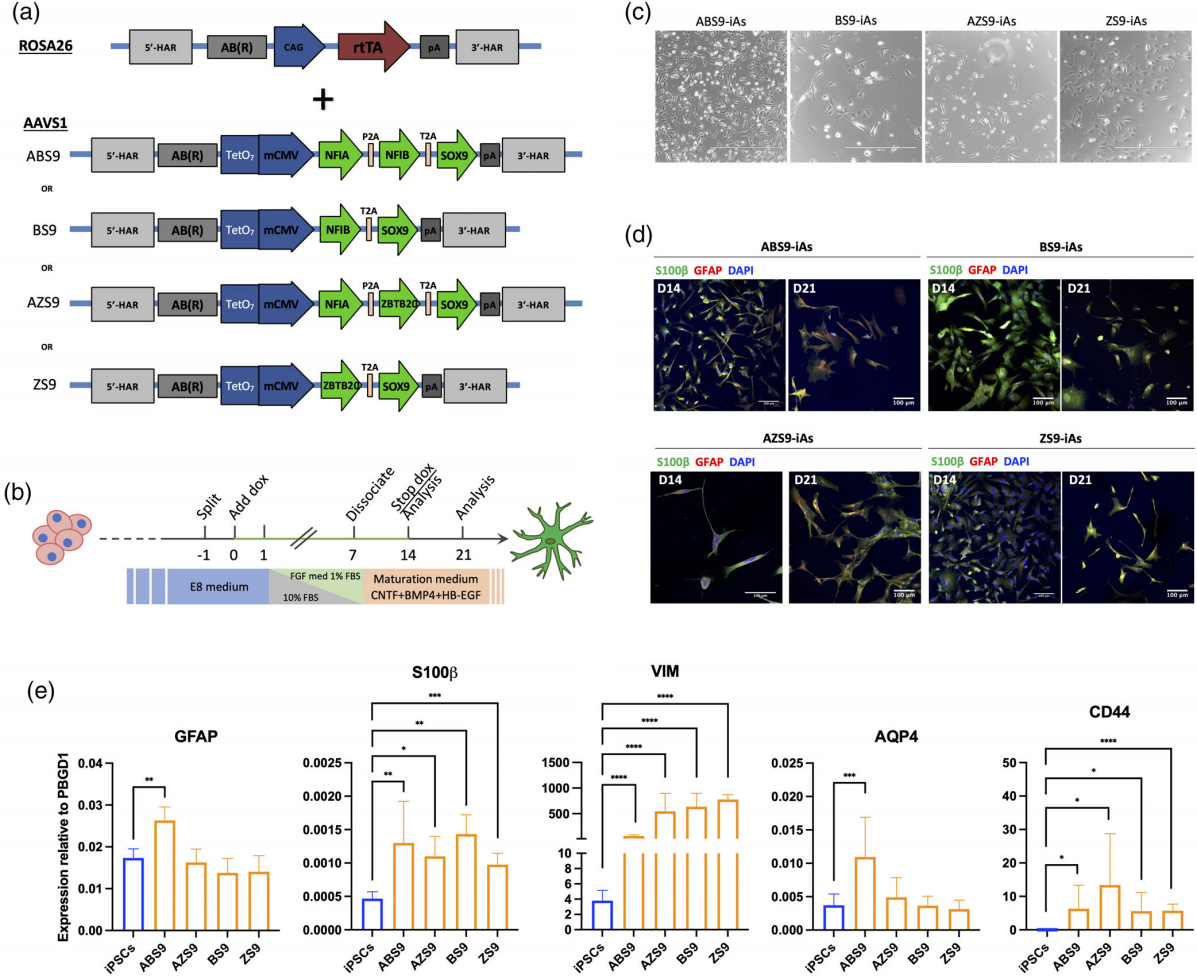
Reprogramming of hiPSCS into iAstrocytes and phenotypical characterization. (a) Schematic depiction of the CAG‐rtTA plasmid used to target the ROSA26 locus and the reprogramming cassettes for the four TFs combinations used to target the AAVS1 locus. (b) Schematic for the experimental setup for generating iAs from hiPSCs. (c) Phase contrast images of the four iAs 1‐week post induction showing flat and/or bi‐polar morphologies. Scale bars = 400 μm. (d) Immunocytochemistry of iAs stained for astrocytic markers GFAP and S100β, 14‐ and 21‐days post induction for all four lines. Scale bars = 100 μm. (e) mRNA expression of astrocyte‐specific markers GFAP, S100β, Vimentin (Vim), AQP4 and CD44 in the four iAs lines. ABS9‐iAs was the only combination to show significant expression of all these markers. *n* = 3 biological replicates, mean ± SD, all values relative to PBGD1. Unpaired student *t* test. *p < .05; **p < .01; ***p < .001; ****p < .0001.

Firstly, we verified successful induction of the four TF cassettes following Dox treatment using RT‐qPCR (Figures 1a and S1a). Time course analysis demonstrated that the expression of the TFs resulted in rapid downregulation of pluripotency genes (e.g., *OCT4*) and that the induced TFs remained expressed until day 14 post induction (Figure S1b). In addition, the respective endogenous genes were also expressed at D14 post induction, indicating that the cassettes had activated endogenous TFs involved in astrocyte identity.

### 3.3 All four TF cassettes induce iAs with astrocyte‐like morphologies and expression of typical markers

Following optimization of culture conditions (Figure 1b), we characterized the resulting reprogrammed cells. At 7 days post treatment initiation, cells started to display morphologies that resembled flat, quiescent astrocytes while others took on bi‐polar shapes with elongated processes (Figures 1c and S1c). The cells were left in culture until days 14 or 21 post‐induction, when they were fixed for ICC. Each of the four TF combinations successfully induced expression of astrocyte‐specific markers, such as GFAP and S100β (Figure 1d). Expression of GFAP was observed as early as day 7 post‐induction for ABS9‐iAs, together with S100β and vimentin (Figure S1c). Expression of GFAP was not shown for iPSCs derived‐NPCs nor for iPSCs (Figure S2). RT‐PCR analysis of iAs on day 14 confirmed the expression of *S100β* and *Vimentin* (*VIM*) in all four lines. However, *GFAP* and *AQP4* were significantly upregulated only in ABS9‐iAs (Figure 1e).

### 3.4 Comparison of transcriptional profiles of iAs derived from different reprogramming cassettes

**FIGURE 2:**
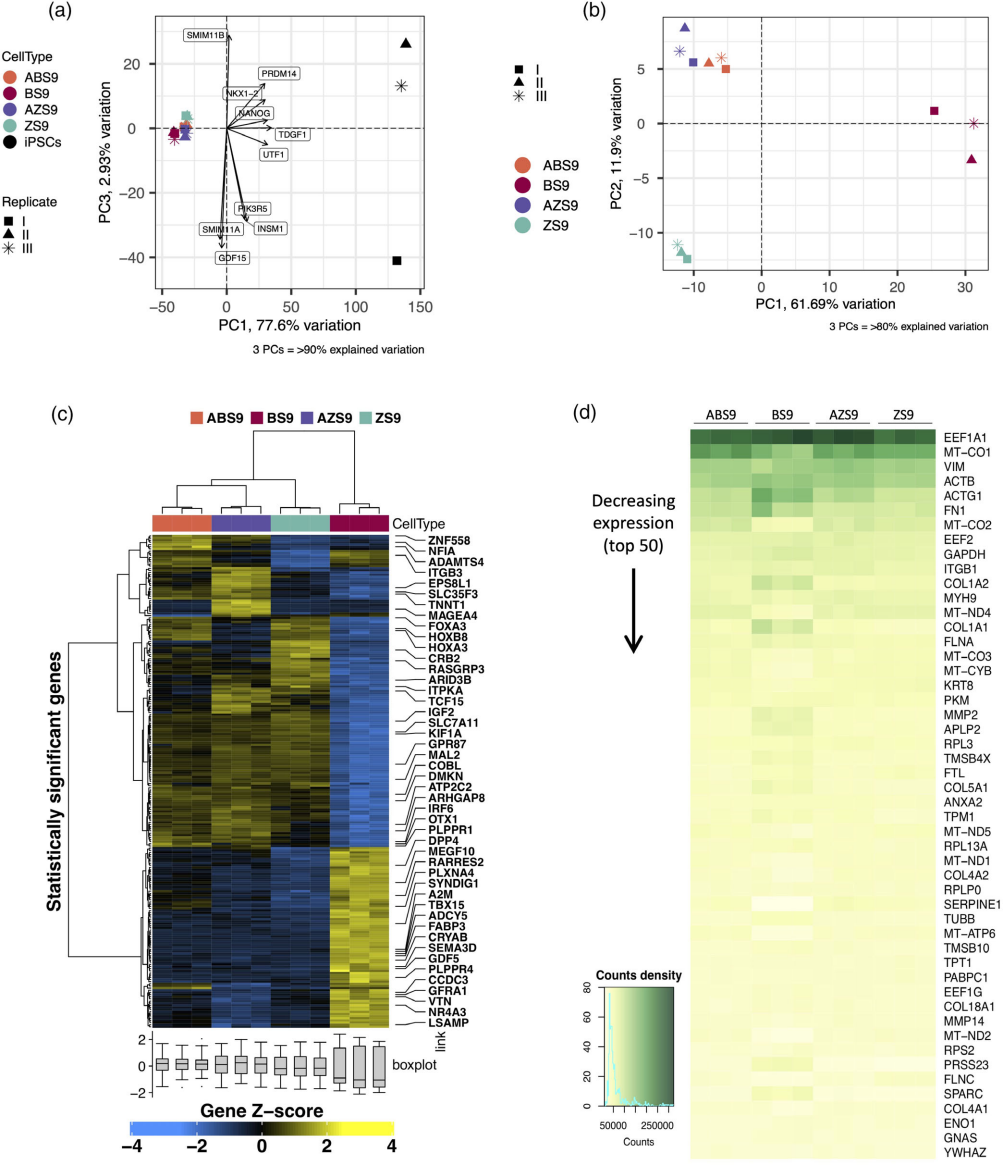
Transcriptomic profiling of iAstrocytes reprogrammed by four distinct cassettes. (a), (b) Principal component (PCA) analysis of the four iAs including (a) and excluding (a) iPSCs controls, showing tight clustering of the four iAs separate from hiPSCs. The percentage of variance explained by the PCAs is indicated between parentheses. (c) Supervised clustering and heatmap analysis of the four iAs lines showing a distinct profile for BS9‐iAs. Genes from each differential expression analysis at Benjamini–Hochberg *Q* ≤ 0.05 and absolute log_2_FC ≥ 2 were included. (d) Heatmap comparison of the top 50 differentially expressed genes between the four iAs lines showing distinct transcriptomic signature for BS9‐iAs.

To characterize the transcriptome of iAs, RNA was extracted at day 14 and subjected to Sanger RNA sequencing. Principal component analysis (PCA) demonstrated tight clustering of the four iAs which were all separated from hiPSCs (Figure 2a). To explore differences between the iAs, a PCA analysis of iAs in the absence of hiPSCs was conducted (Figure 2b). This demonstrated tight clustering of biological repeats into three groups: BS9‐iAs were separated from the ABS9‐iA/AZS9‐iA cluster by principal component 1 (61.69% variation) and ZS9‐iAs were separated by principal component 2 (11.9% variation). Supervised cluster analysis separated BS9‐iAs from the other iAs, which also displayed the highest number (179) of differentially expressed genes (DEG) (Figures 2c and S3). Substituting NFIB with ZBTB20 in ABS9‐iAs versus AZS9‐iAs was associated with only 12 DEGs. Overall, transcriptomic differences between iAs were limited, and mostly related to differences in BS9‐iAs.

### 3.5 NFIB‐SOX9 induces a distinct transcriptomic profile

**FIGURE 3:**
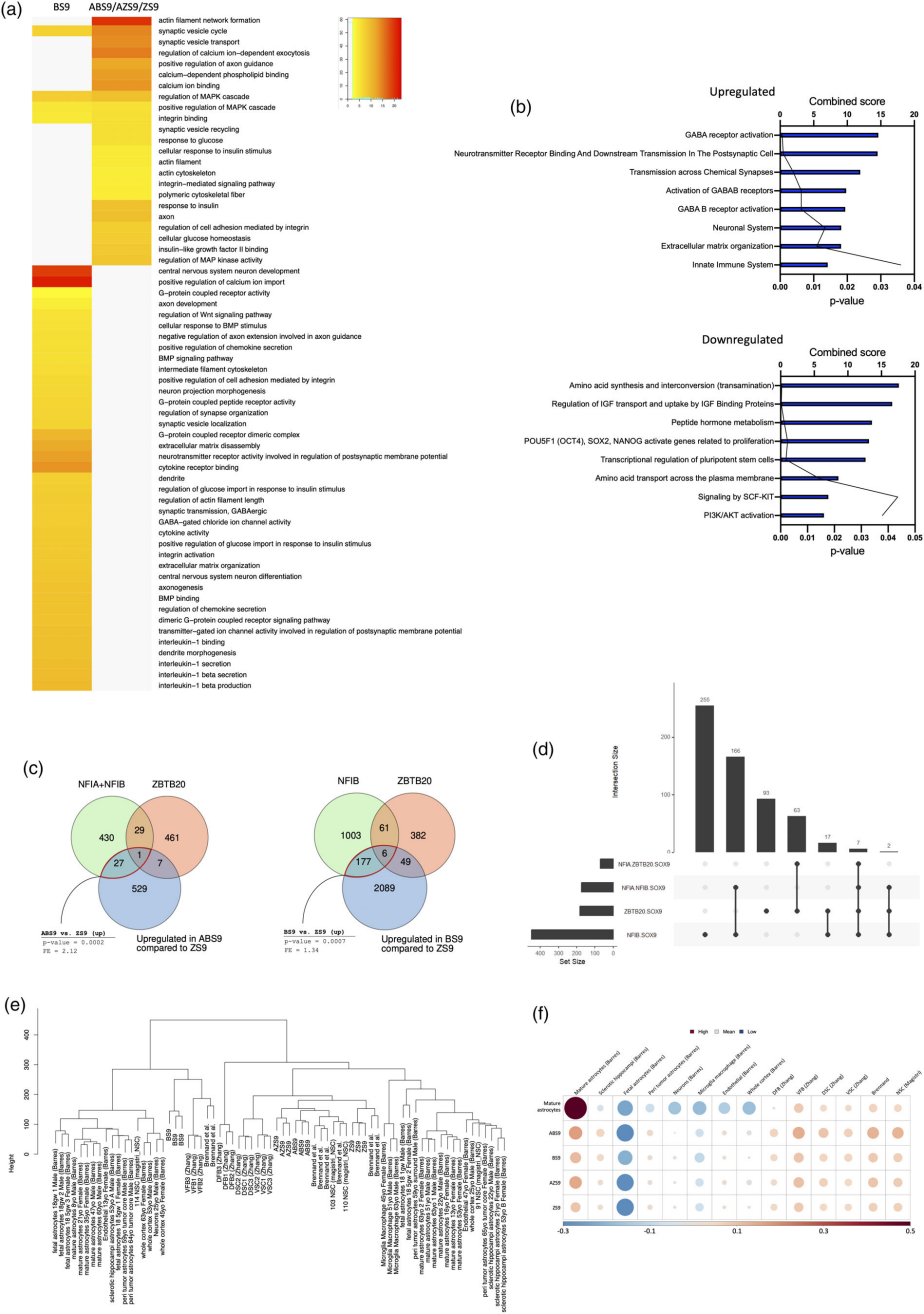
Comparison to published datasets of human astrocytes and gene regulatory network (GRN) analysis. (a) Heatmap comparison of gene ontology (GO) terms for BS9‐iAs versus ABS9/AZS9/ZS9‐iAs, demonstrating upregulation of astrocytic related GO‐terms for all four lines. (b) Bar and line plots depicting signaling pathways upregulated and downregulated in BS9‐iA versus ABS9/AZS9/ZS9‐iAs comparison, demonstrating upregulation of astrocytic related pathways for all four lines. (c) Venn diagrams showing overlap of putative TF binding sites based on their known motifs with TSS‐proximal regions of open chromatin in astrocytes. Based on ATAC‐seq dataset from (M. Song et al., 2019). (d) Upset plots comparing the four TF combinations to find unique and shared direct targets. (e) Hierarchical clustering comparing our RNA‐sequencing data from the four iAs lines to published datasets of human astrocytes. Based on (Bradley et al., 2019; Magistri et al., 2016; TCW et al., 2017; Zhang et al., 2016). All four iAs expressed signatures of mature astrocytes. (f) Cell type enrichment represented by a single value per sample sub‐group plot. DFB, dorsal forebrain; DSC, dorsal spinal cord; VFB, ventral forebrain; VSC, ventral spinal cord.

To further investigate differences in gene expression between iAs derived from different reprogramming cassettes, a heatmap comparison of the top 50 DEGs was conducted. As outlined above, BS9‐iAs were characterized by a distinct transcriptomic signature (Figures 2c and 3d). Hence, the subsequent analysis was focused on the comparison of transcriptomic profiles between BS9‐iAs versus ABS9‐, AZS9‐, and ZS9‐iAs. Gene‐ontology (GO) enrichment analysis revealed GO‐terms related to G protein‐coupled receptors (GPCRs), immune response (cytokine activity) and central nervous system (CNS) development in BS9‐iAs (Figure 3a). The first two represent astrocytic features: GPCRs are associated with calcium signaling (Ward, 2004) and astrocytes are involved in neuroinflammation. In addition, several GO‐terms related to synapse function (mainly GABA), calcium signaling and MAPK signaling were also detected, suggesting features of maturity. Similarly, GO‐terms enriched in ABS9‐, AZS9‐, and ZS9‐iAs included calcium ion binding, regulation of MAPK cascade and synapse related GO‐terms, as well as actin cytoskeleton and insulin response related terms. Pathway analysis supported the above findings and confirmed upregulation of pathways related to synapses, GABA receptors and the innate immune system. In contrast, pathways related to pluripotent stem cells were downregulated (Figure 3b). In BS9‐iAs, up‐regulation of the Wnt signaling pathway was detected, which has been previously associated with repression of astrogliogenesis (Sun et al., 2019).

### 3.6 Distinct astrocytic features were associated with specific TF combinations

To further investigate differences between iAs generated by the distinct TF combinations, we conducted pairwise comparisons of DEGs and TFs (Figures S4 and S5). Previous studies demonstrated the importance of NFIA for both astrocyte differentiation and function; it is also considered as the main regulator of the “gliogenic switch” (Deneen et al., 2006; Huang et al., 2020; Kang et al., 2012; Tchieu et al., 2019; Tiwari et al., 2018). Reprogramming cassettes containing NFIA in ABS9‐iAs and AZS9‐iAs resulted in upregulation of several DEGs compared to BS9‐iAs and ZS9‐iAs, for example, the transcriptional regulators *MSC*, *ZNF558*, and *NFIA*. Exchange of NFIB with ZBTB20 in ABS9‐iAs versus AZS9‐iAs only resulted in 12 DEGs (Figure S3). When comparing BS9‐iAs to ZS9‐iAs, both lacking NFIA in their reprogramming cassette, the number of DEGs increased to 153. Of note, only ABS9‐iAs upregulated the expression of the mature astrocytes markers *AQP4* and *HMGN3*, the latter of which is known to play a critical role in the differentiation of astrocytes (Nagao et al., 2014).

We next sought to assess the extent to which the overexpressed TFs drive the transcriptomic differences observed between the different combinations through their putative direct targets. To infer candidate direct targets of these TFs in astrocytes we overlapped putative TF binding sites based on their known motifs with transcription start site (TSS) proximal regions of open chromatin in astrocytes. This was done for two independent ATAC‐seq datasets (Donohue et al., 2022; M. Song et al., 2019). Based on the analysis, 28 TFs are putatively directly targeted by NFIA and/or NFIB using the Song et al. data and are also upregulated (2.1‐fold enrichment, *p* = .0002) in ABS9‐iAs in comparison to ZS9‐iAs, which lack NFIA/B in their cassette. This is consistent with direct action of one or both these TFs on their putative targets. One hundred eighty‐three TFs are putatively directly targeted by NFIB using the Song et al. data and upregulated in BS9 versus ZS9 in dataset 2 (1.34‐fold enrichment; *p* = .0007) where BS9 has NFIB (and ZS9 does not). The Donohue et al. dataset yielded fewer putative direct targets and the corresponding comparisons were nominally significant but did not pass the multiple testing correction. However, the fold enrichments for these same comparisons were nearly identical to those seen using the Song et al. Taken together, this suggests the promoter‐binding activating effects of NFIB alone and in combination with NFIA significantly contribute to the transcriptomic differences in BS9 and ABS9. (Figures 3c,d and S6a).

### 3.7 Do all four TF combinations lead to similar stages of astrocyte maturity?

Maturity is of critical importance and is a general challenge as far as iPSC‐derived cells are concerned. To further understand the differences between iAs derived from different reprogramming cassettes, we compared the transcriptome of iAs with previously published datasets of mature human astrocytes (Bradley et al., 2019; TCW et al., 2017; Zhang et al., 2016). This analysis revealed that the transcriptomic signatures of the four iAs were similar to mature astrocytes (Figures 3e,f, S6b, and S6c), with ABS9‐ and ZS9‐iAs being the closest to the transcriptional profiles encoding astrocyte maturity.

### 3.8 Do transcriptomic profiles of the four iAs resemble regional differences in human astrocytes?

Recent studies highlight regional differences between astrocytes with regards to function and transcriptomic profiles (Bradley et al., 2019; Herrero‐Navarro et al., 2021). We therefore sought to compare the transcriptomic profiles of iAs with astrocytes from different brain regions. Hierarchical cluster analysis showed a closer association of ABS9‐ and BS9‐iAs to forebrain astrocytes, whereas AZS9‐ and ZS9‐iAs clustered with spinal cord‐derived astrocytes (Figure 3e). Depending on the TF combination used, the iAs resembled cerebral cortex and midbrain astrocytes based on a dataset from Brennand et al. (TCW et al., 2017). It should be pointed that all four iAs were distinct from neurons or other glial cells (Figure S7). Finally, we calculated gene enrichment with regards to regional astrocyte identity by comparison to published human regional astrocyte datasets (Figure 3f). Specifically, we defined a signature for mature astrocytes from Barres et al. and each of dorsal/ventral forebrain and dorsal/ventral spinal cord from Zhang et al. This analysis revealed ABS9‐iAs to be most closely associated with mature, ventral forebrain astrocytes.

### 3.9 Differential activation of iAs in response to IL‐1β

**FIGURE 4:**
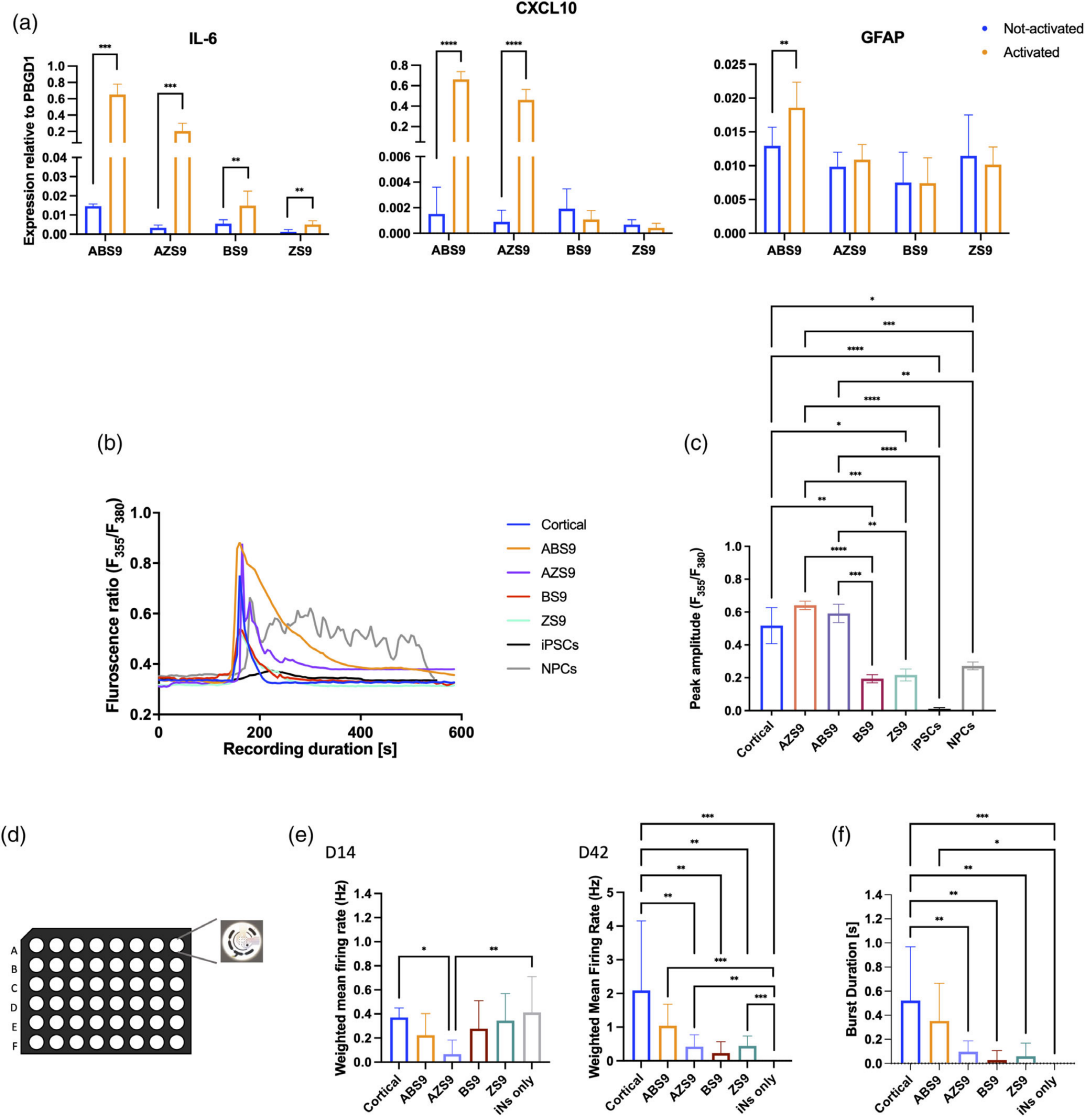
Functionality of the human iAs. (a) Fold increase in IL‐6, CXCL10 and GFAP expression after 8 h of IL‐1β treatment was shown mainly for ABS9‐iAs. *n* = 4–6 biological replicates, mean ± SD, all values relative to PBGD1, unpaired student *t* test. (b) Representative sample traces of Ca^2+^ signals triggered by ATP in the four iAs lines (ABS9, AZS9, BS9, ZS9), in hiPSCs, in NPCs, and in rodent cortical astrocytes (Cortical). All four lines showed traces of Ca^2+^ elevation in response to ATP. (c) Histogram of the peak amplitude triggered by ATP in the four iAs lines (ABS9, AZS9, BS9, ZS9), in hiPSCs, in NPCs, and in rodent cortical astrocytes (Cortical). Peak amplitudes were significantly higher for ABS9‐ and AZS9‐iAs. *n* = 3 biological replicates, total of 30–50 cells were measured for each line; mean ± SEM, One‐way analysis of variance (ANOVA), Dunnett's multiple comparisons test. (d) Schematic representation of the MEA recording. (e) Bar plots comparing the weighted firing rate of human iNs cultured with each one of the four iAs lines (ABS9, AZS9, BS9 or ZS9), rodent cortical astrocytes (Cortical) or iNs alone (iNs only) at 14‐, 21‐, and 42‐days post iNs induction. *n* = 8, data from 128 electrodes per condition, mean ± SD, One‐way ANOVA, Dunnett's multiple comparisons test. (f) Bar plots comparing the bursting duration of human iNs between the four iAs lines, rodent cortical astrocytes (Cortical) and iNs alone (iNs only) at 42‐days post iNs induction. *n* = 8, data from 128 electrodes per condition, mean ± SD, One‐way ANOVA, Dunnett's multiple comparisons test. *p < .05; **p < .01; ***p < .001; ****p < .0001.

We next sought to investigate how the transcriptomic differences of iAs reprogrammed with the four different TF‐cassettes relate to cell function. An important role of astrocytes is the regulation of the inflammatory response. We therefore exposed iAs to IL‐1β, a pro‐inflammatory cytokine that is known to induce a reactive astrocyte phenotype (Lee et al., 1993). RT‐qPCR assessment of cytokines (*IL‐6*, *CXCL10*) and *GFAP* showed that iAs whose reprogramming cassette included NFIA (ABS9 and AZS9) had more pronounced responses to IL‐1β than those lacking NFIA (Figure 4a). While *IL‐6* was upregulated in all four lines following activation, *CXCL10* was significantly upregulated only in ABS9‐ and AZS9‐iAs. Moreover, *GFAP* upregulation post treatment was observed only for ABS9‐iAs.

### 3.10 Differences in Ca^2+^ signaling between iAs derived from different reprogramming cassettes

Astrocytes are known to undergo elevations in intracellular calcium levels in response to physiological and pathological stimuli (Newman, 1997; Parpura et al., 1994). To assess whether our iAs display differences with regards to ATP‐induced calcium waves, calcium imaging using fura‐2/AM, a ratio‐metric calcium indicator, was conducted. All four iAs responded to the addition of ATP to the medium and showed traces of Ca^2+^ elevation that had similar pattern to those of primary rodent astrocytes (Figure 4b). Peak amplitudes triggered by ATP were comparable to that of primary astrocytes in ABS9‐ and AZS9‐iAs, but significantly lower in BS9‐ and ZS9‐iAs (Figure 4c). However, peak calcium amplitudes displayed by BS9‐ and ZS9‐iAs were not significantly different from those in iPSCs derived NPCs, suggesting a progenitor‐like state. Human iPSCs controls did not display Ca^2+^ changes in response to ATP.

### 3.11 iAs support electrophysiological activity of iNs

Astrocytes play an important role at the synapse coordinating metabolism and maturity (Baldwin & Eroglu, 2018). In vitro, neurons require the presence of rat astrocytes to form functional synapses (Tourigny et al., 2019; Zhang et al., 2013). To establish whether iAs were able to induce synapse maturation, cells were co‐cultured with OPTi‐OX human induced neurons (iNs) (Pawlowski et al., 2017; Tourigny et al., 2019) on MEAs. The electrophysiological activity of the iNs was monitored over a period of 42 days. iNs cultured with iAs were compared to iNs cultured with primary rat cortical astrocytes and with iNs monocultures. MEA recordings confirmed that iNs cultured in the presence of iAs and primary astrocytes undergo synaptic maturation. This was indicated by an increase in the weighted mean firing rate. However, not all combinations induced the same level of maturity. By day 42, ABS9‐iAs were associated with the highest firing rate. In addition, ABS9‐iAs co‐cultures displayed burst firing activity, which were comparable to co‐cultures of iNs with rat astrocytes at day 42 (Figure 4e).

### 3.12 ABS9‐iAs survive and integrate following transplantation into the CNS

**FIGURE 5:**
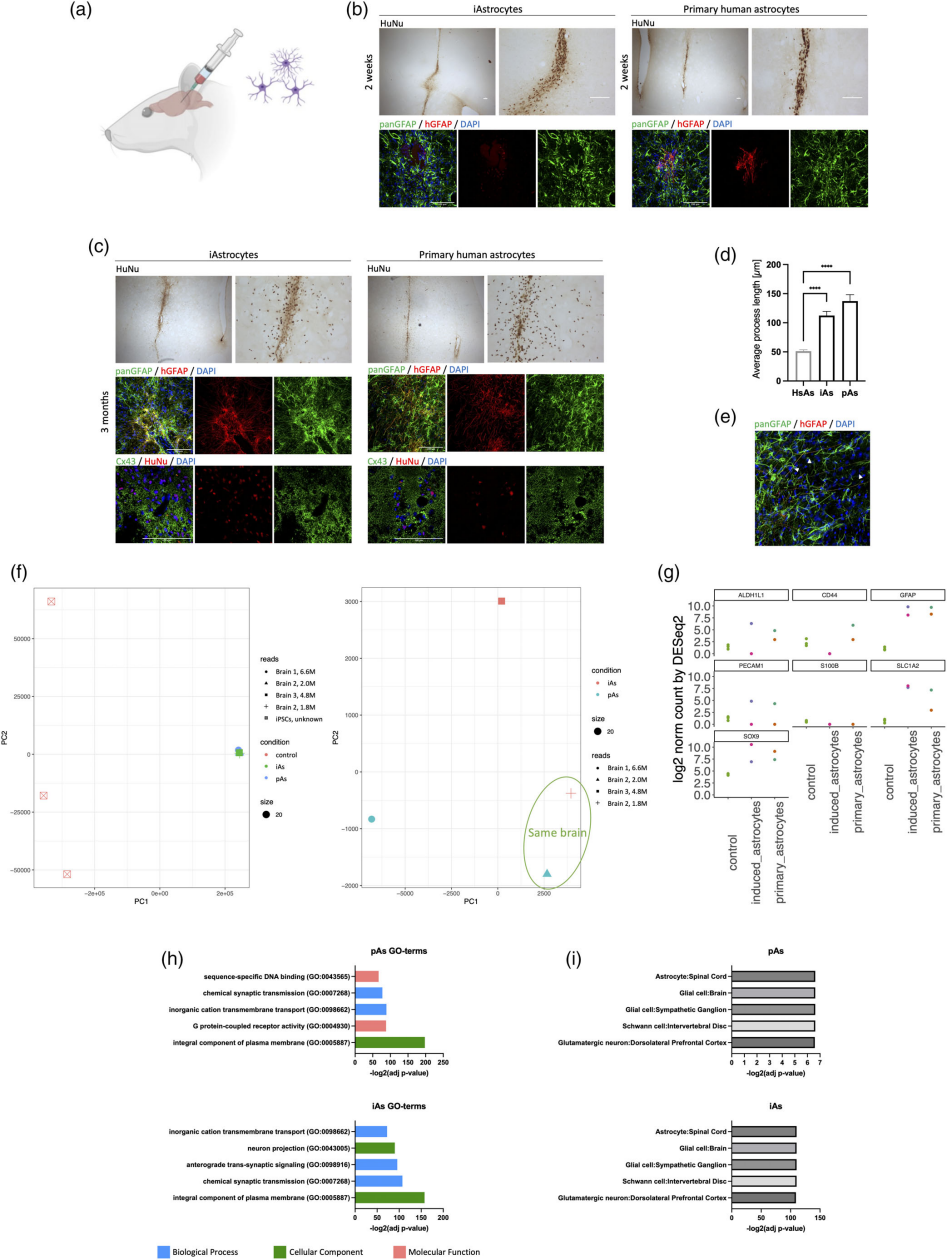
Integration into a rat brain model. (a) Schematic drawing of the transplantation into rat brains. Rats were intracerebrally injected with iAs (left hemisphere) or pAs (right hemisphere). Created with BioRender.com. (b) Survival of transplanted ABS9‐iAs at 2 weeks. 3,3′‐Diaminobenzidine (DAB) staining and immunohistochemistry for hGFAP, panGFAP, and DAPI in the lesion site. HuNu, Human nuclei. Scale bars = 100 μm. (c) Long‐term survival and integration of ABS9‐iAs at 3 months. DAB staining and immunohistochemistry for hGFAP, panGFAP, Cx43, and DAPI in the lesion site. HuNu, Human nuclei. Scale bars = 100 μm. (d) Average process length comparison between iAs, primary astrocytes (pAs) and host rodent astrocytes (HsAs). *n* = 30 per treatment group, mean ± SEM, One‐way analysis of variance (ANOVA), Tukey's multiple comparisons test. (e) Immunohistochemistry for hGFAP, panGFAP, and DAPI in the lesion site. Arrowheads point to human iAs processes. Scale bar = 100 μm. (f) PCA analysis of the transplanted primary and induced astrocytes including (left) and excluding (right) hiPSC controls. iAs and pAs from the same brain (brain 2, right PCA, “+” and “Δ”) cluster closer together than those from different brains. (g) Number of counts for astrocytic markers in each sample (iAs or pAs) compared to control iPSCs. (h) GO‐term analysis based on Enrichr, showing up regulation of similar terms in both pAs and iAs. (i) Cell type analysis based on Enrichr, showing that both pAs and iAs resemble astrocyte/glia like cells. ***p < .001.

Based on the transcriptomic data and functional properties outlined above, all four TFs combinations led to cells with astrocyte‐like characteristics. ABS9‐iAs most closely resembled mature forebrain astrocyte identity, therefore, this line was chosen for further in vivo work. To assess long‐term stability of ABS9‐iAs and their ability to integrate into the CNS, cells were transplanted into the striatum of immunocompromised NIH‐*Foxn1rnu* nude rats—an approach similar to that used by us and others (W. L. Kuan et al., 2007; Proschel et al., 2014). As a control, commercially available primary human cortical astrocytes were used. Two weeks following transplantation animals were sacrificed. Stereologic analysis based on anti‐human nuclear antigen IHC enabled quantification of the transplanted cells. At 2 weeks post‐injection, both iAs and primary human astrocytes survived the transplantation with similar viability (3%–5%). In both cases, transplanted cells remained close to the needle tracts (Figure 5b). IHC staining with antibodies specific to human GFAP revealed that primary human astrocytes expressed higher levels of GFAP at this time point. At 3 months post‐transplantation, brain sections stained with human nuclear antigen demonstrated that iAs and primary human astrocytes had migrated up to several millimeters away from the transplantation site (Figure 5c). IHC for human GFAP revealed morphological maturation of iAs after 3 months in vivo: iAs exhibited long slender processes resembling those seen on primary human astrocytes, which were distinct from the native rodent cells with fewer and blunter processes (Figure 5d,e). IHC staining for Cx43, a major constituent of astrocytic gap junctions, demonstrated multiple puncta intimately surrounding human cells. This suggests that the iAs may have connected with the astrocytic syncytium of the host.

To further analyze transplanted iAs, bulk RNA‐seq was conducted 3 months post transplantation of brains with primary or iAs. Human sequences were detected using SARGASSO (Qiu et al., 2018). PCA analysis of transplanted cells and including RNA‐seq profiles of hiPSCs demonstrated tight clustering of human induced and primary astrocytes (Figure 5f, left panel). Removal of hiPSC‐RNA‐profiles enabled to tease out more subtle differences between primary astrocytes and iAs. This demonstrated that iAs and pAs from the same brain cluster closer together than those from different brains (Figure 5f, right panel).

We next compared the number of sequencing counts for astrocytic markers, including ALDH1L1, CD44, GFAP, PECAM1, S100β, SLC1A2, and SOX9 in each sample (iAs or pAs) and in control iPSCs (Figure 5g). This demonstrated a close resemblance in expression patterns between iAs and pAs. GO‐term analysis demonstrated up regulation of similar terms in both pAs and iAs (Figure 5h), and cell type analysis demonstrated that both pAs and iAs resembled astrocyte/glia like cells (Figure 5i).

In conclusion, these data demonstrate that ABS9‐iAs were able to integrate and mature following transplantation into the rat brain in vivo.

## 4 DISCUSSION

This study aimed to investigate four different TF combinations (ABS9, BS9, AZS9, ZS9) that encode astrocyte identity. Highly efficient reprogramming of hiPSCs was achieved using OPTi‐OX, a dual safe‐harbor gene targeting strategy (Pawlowski et al., 2017). This approach overcomes gene silencing and is able to successfully generate iNs, myocytes and oligodendrocytes with purities approaching 100%, without the need for sorting steps.

ICC characterization of the reprogrammed cells established that all four TF combinations resulted in the expression of major astrocytic markers, including vimentin, S100β and GFAP. Loss of pluripotency was confirmed by rapid downregulation of associated genes. Comparison of the transcriptomic profiles of different iAs demonstrated subtle variations in gene expression. Comparison of RNA signatures with ex vivo primary astrocytes from different brain regions indicated similarities with mature brain astrocytes; in particular, ABS9‐iAs resembled ventral forebrain astrocyte profiles.

To assess their functional response, the iAs were exposed to a pro‐inflammatory cytokine IL‐1β. Cells reprogrammed with NFIA (ABS9 and AZS9) demonstrated more pronounced cytokine responses than BS9‐iAs and ZS9‐iAs. Calcium imaging demonstrated the ability of all iAs to generate intracellular calcium signals, however, the amplitude of the response was specific to the TF combination used to reprogram iPSCs. Therefore, functional data confirmed that ABS9‐iAs most closely resembled mature cortical astrocytes, this was further supported by an enhanced electrical maturation of iAs‐iNs co‐cultures on MEAs.

To assess long term phenotypic stability and in vivo integration, ABS9‐iAs were transplanted into the brain of immunocompromised rats. Histological assessment up to 3 months post‐transplantation demonstrated successful long‐term survival, migration, and integration comparable to primary human cortical astrocytes, albeit morphological maturation of iAs was delayed.

Our experiments demonstrate that (1) all four reprogramming cassettes induced stable attractor states that converged on a general astrocyte identity. (2) However, different reprogramming cassettes activated distinct GRNs that resulted in subtle differences in gene expression of iAs. (3) These transcriptomic differences were associated with significant functional differences. (4) Transplantation of ABS9‐iAs, which most closely matched the profile of human primary cortical astrocytes, led to long‐term survival and integration of cells into the host rodent CNS.

### 4.1 Selection of TFs and their effects on iAs properties

Candidate TFs for reprogramming hiPSCs into an astrocytic phenotype were selected from the literature taking into account previous reprogramming protocols and the contribution of TFs to astrocyte development (Caiazzo et al., 2015; Canals et al., 2018; Li et al., 2018). All four combinations of TFs, in our study, were able to induce cells displaying the expected astrocyte morphology and astrocytic marker expression profile. Transcriptomic analysis of the resulting iAs demonstrated subtle differences with a modest number of differentially regulated genes. This indicates a strong attractor state encoding astrocyte identity.

#### 4.1.1 SOX9

During embryonic development, SOX9 plays a critical role in directing the cells into a glial fate (Stolt, 2003). It has been implied in the gliogenic switch and shown to directly regulate NFIA expression; together SOX9 and NFIA direct astrocytes differentiation and maturation (Kang et al., 2012). In the context of cell reprogramming, SOX9 has been used in combination with other genes to successfully induce astrocyte identity (Caiazzo et al., 2015; Canals et al., 2018; Li et al., 2018). SOX9 formed part of all TF programs tested in the present study.

#### 4.1.2 NFIA

Recent evidence suggests that NFIA is an important factor that directs astrocyte differentiation by switching the chromatin state of its target regulatory elements from primed to active (Tiwari et al., 2018). iAs reprogrammed with NFIA were functionally more mature, displaying a more pronounced cytokine response with upregulation of *CXCL10* in addition to *IL‐6*, and larger peak amplitudes of Ca^2+^ signaling. Our transcriptomic analysis further corroborates this view as multiple astrocytic maturation markers were upregulated in NFIA‐induced iAs.

#### 4.1.3 ZBZT20 and NFIB in the absence of NFIA

After the gliogenic switch occurs, NFIB helps drive astrocytic maturation (Deneen et al., 2006; Matuzelski et al., 2017), while ZBTB20 was found to promote astrocytogenesis and to regulate astrocyte specification (Nagao et al., 2016). ZBTB20 was included due to its important role in development that has not yet been investigated in the context of astrocyte reprogramming. Principal component analysis of RNA‐Sequencing data revealed that cells reprogrammed with TF cassettes that did not include NFIA clustered away from other iAs. Principal component 1 separated BS9‐iAs accounting for 61.69% of the variation, while principal component 2 separated ZS9‐iAs based on 11.0% of variation. Hence, BS9‐RNA profiles showed the largest differences between iAs, including the greatest number of differentially regulated genes. A potential explanation for the larger discrepancy of BS9 is provided by findings that ZBTB20 may act as an upstream regulator of NFIA.

### 4.2 Do iAs demonstrate comparable levels of maturity?

RNA sequencing demonstrated robust upregulation of maturation‐associated genes in all iAs, and down‐regulation of pluripotency signatures and early astrocyte‐enriched genes. Nevertheless, differences with regards to maturity were detected. ABS9‐iAs and AZS9‐iAs profiles most closely resembled RNA‐signatures of mature astrocytes. Hence, some of the transcriptomic and phenotypic differences observed may be related to the maturity of the resulting iAs.

### 4.3 Functional differences between iAs

**FIGURE 6:**
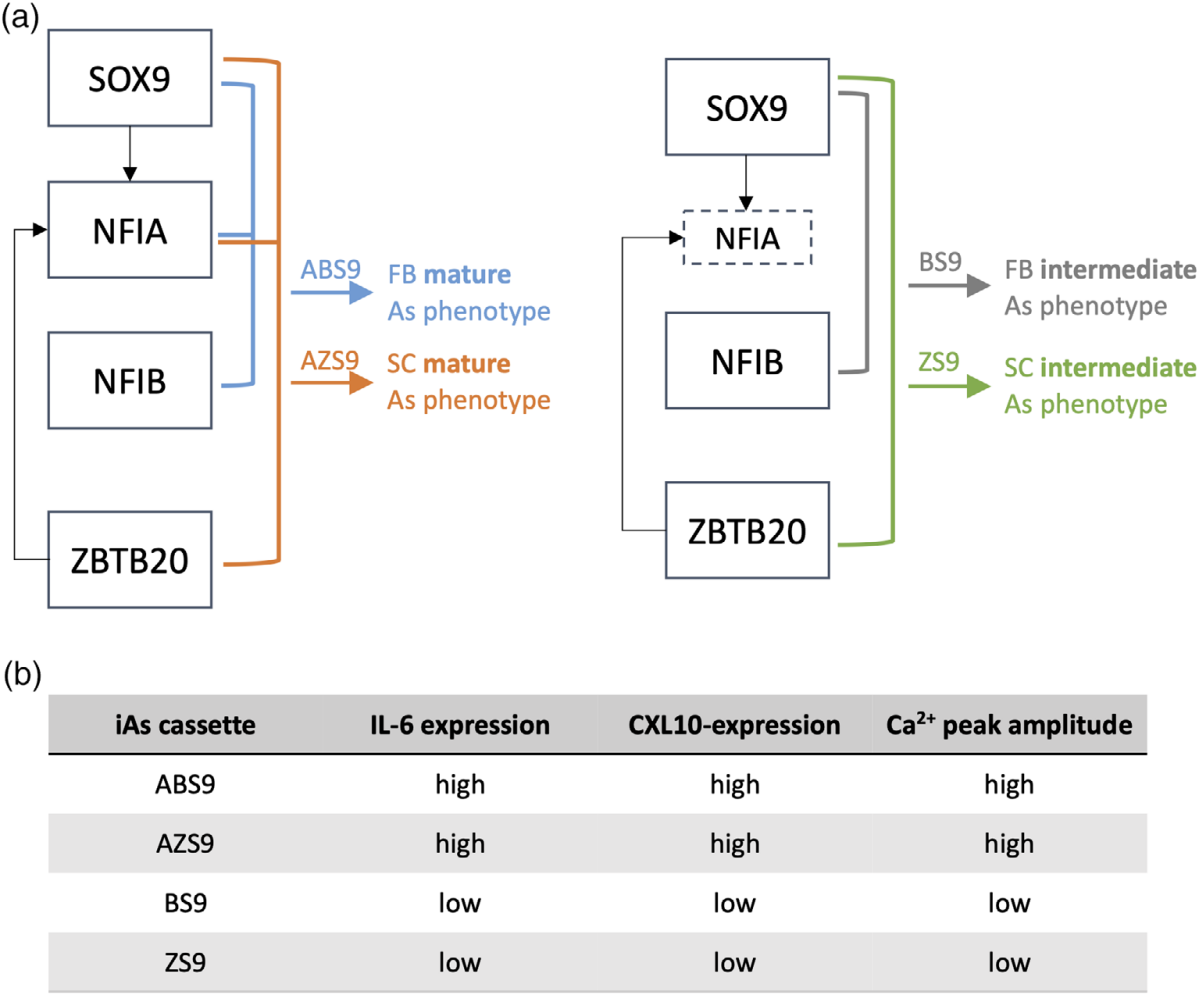
Summary of the results. (a) Suggested model to generate iAs according to maturity and/or astrocytic region using different transcription factor (TF) combinations. As, astrocytes; FB, forebrain; SC, spinal cord. (b) Summary of functional differences between the four iAs lines.

The resulting iAs displayed profound differences with regards to their functional properties. iAs induced with TF cassettes containing NFIA demonstrated increased release of cytokines upon pro‐inflammatory assay and larger peak amplitudes in Ca^2+^‐signaling (also see summary in Figure 6).

Astrocytes can be activated in response to pro‐inflammatory stimuli, to which they respond by releasing cytokines and upregulating GFAP (Anderson et al., 2014). TF cassettes including NFIA yielded iAs that displayed increased activation following IL‐1β assay. In keeping with the notion that ABS9‐iAs represent the most mature phenotype, these cells exhibited the most pronounced expression of GFAP. Nevertheless, regional differences exist with regards to astrocyte activation that could have accounted for differential iAs response to an inflammatory stimulus (Morga et al., 1998; Wong et al., 1999).

In addition, astrocytes respond to a wide range of physiological and pharmacological stimuli with elevations in intracellular Ca^2+^ concentration ([Ca^2+^]_i_) (Guthrie et al., 1999; Salter & Hicks, 1994). ATP is one of the extracellular signaling molecules that activates P2 purinoceptor family, which consists of metabotropic P2Y receptors (P2YR) and ionotropic P2X receptor (P2XR), and elevates astrocytic [Ca^2+^]_i_ (Burnstock & Verkhratsky, 2012; James & Butt, 2002; Salter & Hicks, 1995). RNA‐sequencing revealed that *P2RY2* was significantly downregulated in the BS9‐ and ZS9‐iAs compared to ABS9‐ and AZS9‐iAs, providing a potential explanation for the higher amplitude of calcium waves in NFIA‐induced cells. Further experiments are required to confirm the functional relevance of *P2RY2*.

### 4.4 Do iAs represent regionally distinct astrocyte identities?

ABS9‐ and ZS9‐iAs both displayed the most mature transcriptomic profiles, but their functional properties were very distinct. This raises the question whether iAs could resemble different subtypes of astrocytes. Astrocytes from various brain regions and even subtypes within the same region have unique signaling properties and immunoreactivity (Batiuk et al., 2020; Morga et al., 1998; and our unpublished data). Comparison of transcriptomic profiles of iAs with datasets from primary astrocytes revealed that ABS9‐iAs clustered more closely to forebrain astrocytes, whereas ZS9‐iAs shared similarity with spinal cord‐derived astrocytes, in addition to ventral forebrain and dorsal forebrain identities (Figure 3d,e). The comparison was limited by the lack of data on primary astrocyte identity. BS9‐iAs were distinctively different from the other three iAs. First, their transcriptomic profile resembled that of ventral forebrain astrocytes, second Ca^2+^ imaging resembled that of NPCs, and third a reduced response to cytokines was observed pointing to an immature phenotype with a different astrocytic region specificity. Taken together, these data suggest a model in which different combinations of TFs lead to different astrocytic regions, which can then be either in an intermediate or a more mature astrocytic stage, as illustrated in Figure 6a.

### 4.5 Transplantation of iAs into rat brains demonstrates long‐term stability and integration

To test long‐term stability and integration of iAs, cells were transplanted into the brains of NIH‐*Foxn1rnu* nude rats. We selected ABS9‐iAs as these displayed the most mature phenotype, both with regards to RNA‐profiles and function. Cells survived in the host brain for at least 3 months and generated GFAP‐expressing cells that were morphologically and transcriptionally indistinguishable from the primary human astrocytes used as a control.

Two weeks post transplantation, iAs were developmentally behind primary human astrocytes, based on morphology and GFAP expression, however, 3 months after transplantation, they reached similar levels of maturity and integration, at which point they were indistinguishable. Both human iAs and primary human astrocyte morphology was distinct from the rat astrocytes, displaying more numerous, longer, and increasingly branched processes, as has been described previously (Oberheim et al., 2009). Expression of connexins indicated the formation of gap junctions and suggest integration of iAs into the host astrocytic syncytium.

RNA‐Seq of human transcripts analysis revealed that after 3 months in vivo, iAs displayed a transcriptomic profile similar to the human primary astrocytes and distinct from hiPSCs (Figure 5f). iAs and primary human astrocytes harvested from a single animal displayed remarkably similar transcriptional profiles. In addition, GO‐terms analysis showed upregulation of synaptic related terms (e.g., chemical synaptic transmission), and cell type analysis suggested that the transplanted cells most closely resembled astrocytes/glia (Figure 5h,i).

Formation of gap junctional network is an important feature of mature astrocytes, and we sought to address the ability of iAs to connect with rat astrocytes. We found punctate connexin43 staining closely surrounding transplanted iAs; however, due to the lack of human‐specific gap junctional markers, it was not possible to assess functional coupling between rat and human cells in the brain.

Engraftment of astrocytes into the brain provides a valuable tool to study the cell autonomous disease mechanisms (Benraiss et al., 2016; Papadeas et al., 2011; Qian et al., 2017) as well as the involvement of specific cell types in memory consolidation and other cognitive functions (Han et al., 2013). In addition, transplantation of astrocyte‐lineage cells is becoming an increasingly recognized therapeutic strategy that yielded promising results in ALS (Izrael et al., 2018; Lepore et al., 2008; Nicaise, 2015), Parkinson's (Proschel et al., 2014; J.‐J. Song et al., 2017), Alzheimer's (Esposito et al., 2016; Hampton et al., 2010), and Huntington's (Benraiss et al., 2016) diseases as well as traumatic injury (Davies et al., 2011; Hastings et al., 2022). However, the question of the long‐term cell stability in a diseased environment and the regional identity of the graft warrants further investigation. The ability to precisely engineer human astrocytes based on cell reprogramming may lend itself to clinical products with enhanced product definition.

### 4.6 Limitations

The main limitations of the present study are the number of TF cassettes tested: given the observed levels of redundancy, we expect that other combinations exist that will be able to induce astrocyte identity. A systematic, combinatorial screen of all TFs that are expressed in astrocytes would provide a comprehensive understanding of the astrocyte attractor state.

In conclusion, this study provides insights into four TF combinations that are necessary and sufficient for reprogramming of hiPSCs into a stable astrocyte identity. It provides novel insights into the attractor state that encodes astrocyte identity and in particular highlights how subtle differences in gene expression are associated with significant differences in cell function. Cell reprogramming enables precision engineering of human cells with regards to cell function and provides a scalable platform for manufacturing of human cells, a fundamental requirement for clinical application of cells.

## AUTHOR CONTRIBUTIONS

Koby Baranes and Mark R. N. Kotter conceived the project and designed the study. Koby Baranes, Nataly Hastings, Saifur Rahman, Joana M. Tavares, Noah Poulin, and Wei‐Li Kuan performed the experiments. Koby Baranes, Nataly Hastings, Saifur Rahman, Najeeb Syed, Meik Kunz, Kevin Blighe, and Grant Belgard analyzed data. Koby Baranes, Nataly Hastings, and Mark R. N. Kotter co‐wrote the manuscript.

## FUNDING INFORMATION

Koby Baranes is supported by the Multiple Sclerosis (MS) Society and bit. bio LTD (https://bit.bio/). Nataly Hastings is supported by the Addenbrooke's Charitable Trust (ACT), Ferblanc Foundation, and the Rosetrees Trust. Wei‐Li Kuan is supported by the Medical Research Council (MR/S005528/1). Mark R. N. Kotter is supported by a NIHR Clinician Scientist Award. Disclaimer: This report is independent research arising from a Clinician Scientist Award, CS‐2015‐15‐023, supported by the National Institute for Health Research. The study is further supported by the Cambridge Brain Injury MIC. The views expressed in this publication are those of the authors and not necessarily those of the NHS, the National Institute for Health Research or the Department of Health and Social Care.

## CONFLICT OF INTEREST STATEMENT

bit. bio holds an exclusive license to OPTi‐OX via Cambridge Enterprise and has supported the work. MRNK is co‐inventor of OPTi‐OX and director and shareholder in bit.bio.

## Supporting information


**DATA S1:** Supporting Information

## Data Availability

The data that support the findings of this study are available from the corresponding author upon reasonable request.
