# Supplementary material for: Transcription factor combinations that define human astrocyte identity encode significant variation of maturity and function
Source: Glia. 2023 Apr 8;71(8):1870–89. doi: 10.1002/glia.24372 (PMC10952910; doi:10.1002/glia.24372)
Supplement: Supplementary file 1 — DATA S1: Supporting Information [file GLIA-71-1870-s001.docx]

**Supporting Information**

**Transcription factor combinations that define human astrocyte identity encode significant variation of maturity and function**

Koby Baranes, Nataly Hastings, Saifur Rahman, Noah Poulin, Joana M. Tavares, Wei-Li Kuan, Najeeb Syed, Meik Kunz, Kevin Blighe, Eleni Patili, T. Grant Belgard, Mark R.N. Kotter

**Supplementary Figures**

**
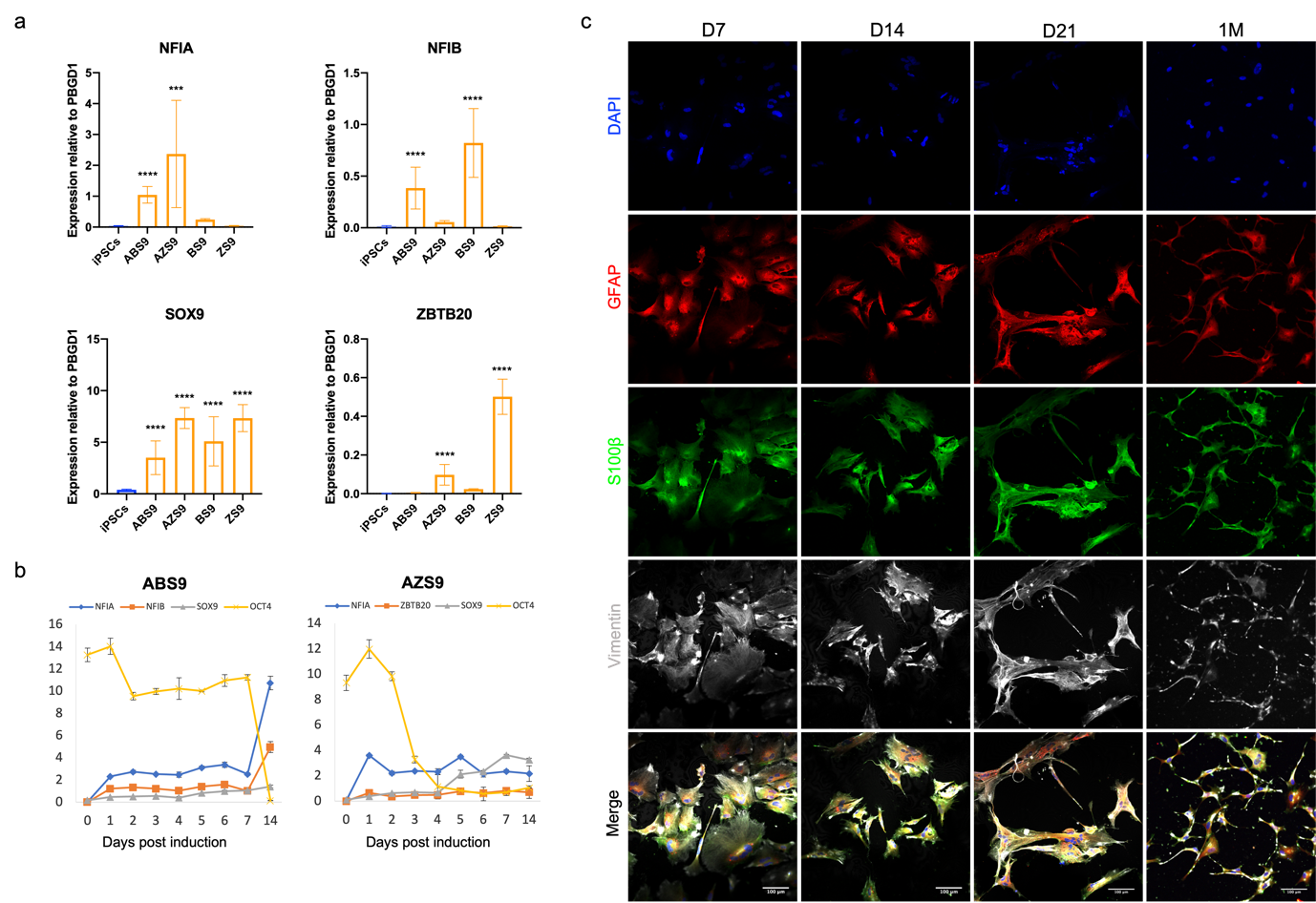
Supplementary Figure S1. iAstrocytes characterization.**

(a) mRNA levels of the 4 transcription factors in each one of the 4 lines. (n = 4-6 biological replicates; mean ± SD; all values relative to PBGD1; Unpaired student t-test).

(b) Representative qPCR analysis demonstrating the time course expression pattern of the defined transcription factors and the pluripotency gene OCT4. (n = 3 biological replicates; mean ± SD; all values are relative to PBGD1).

(c) Immunocytochemistry in ABS9-iAs for GFAP, S100β and vimentin at D7, D14, D21 and 1-month post induction. Scale bars = 100µm.

***p<0.001, ****p<0.0001


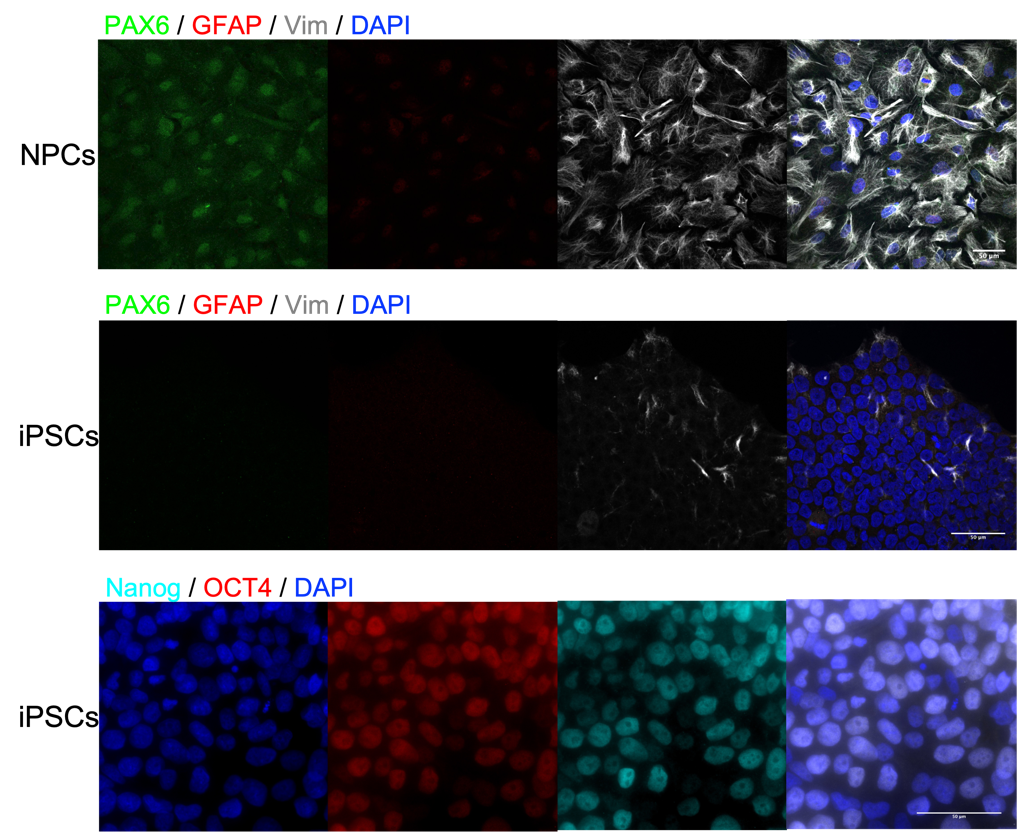


**Supplementary Figure S2. Validation of astrocytic markers in human iPSCs and NPCs**

Immunocytochemistry in human iPSCs-derived NPCs (upper panel) and human iPSCs (middle and lower panels) for GFAP, vimentin, PAX6, OCT4 and Nanog. Scale bars = 50µm.


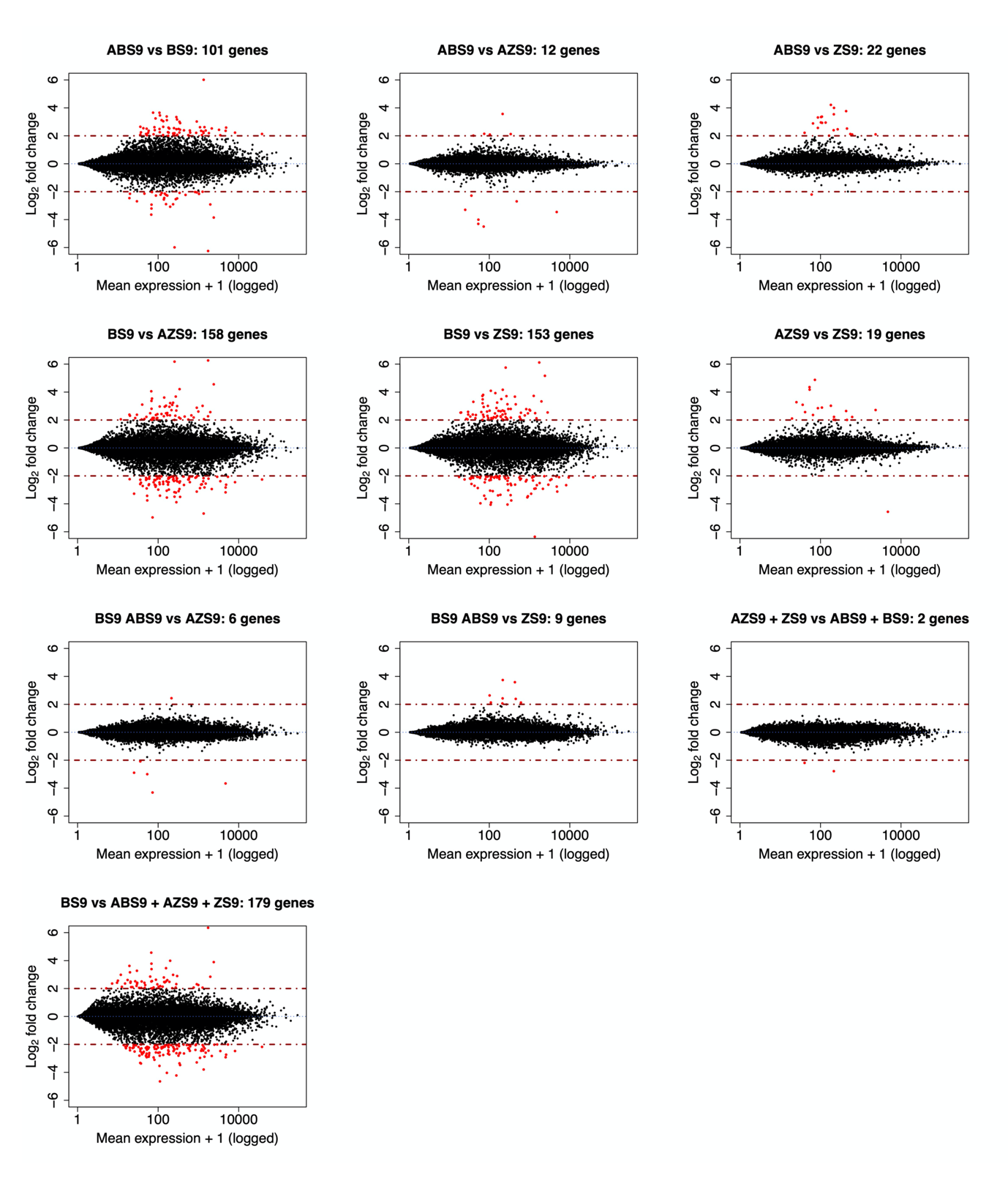


**Supplementary Figure S3**. **MA plot comparing gene expression profiles.**

Different comparisons were performed to find genes significantly different between the 4 iAs lines. Genes passing Benjamini-Hochberg Q≤0.05 and absolute log_2_FC≥2 were highlighted in red. Number of genes significantly different is indicated in each comparison title.


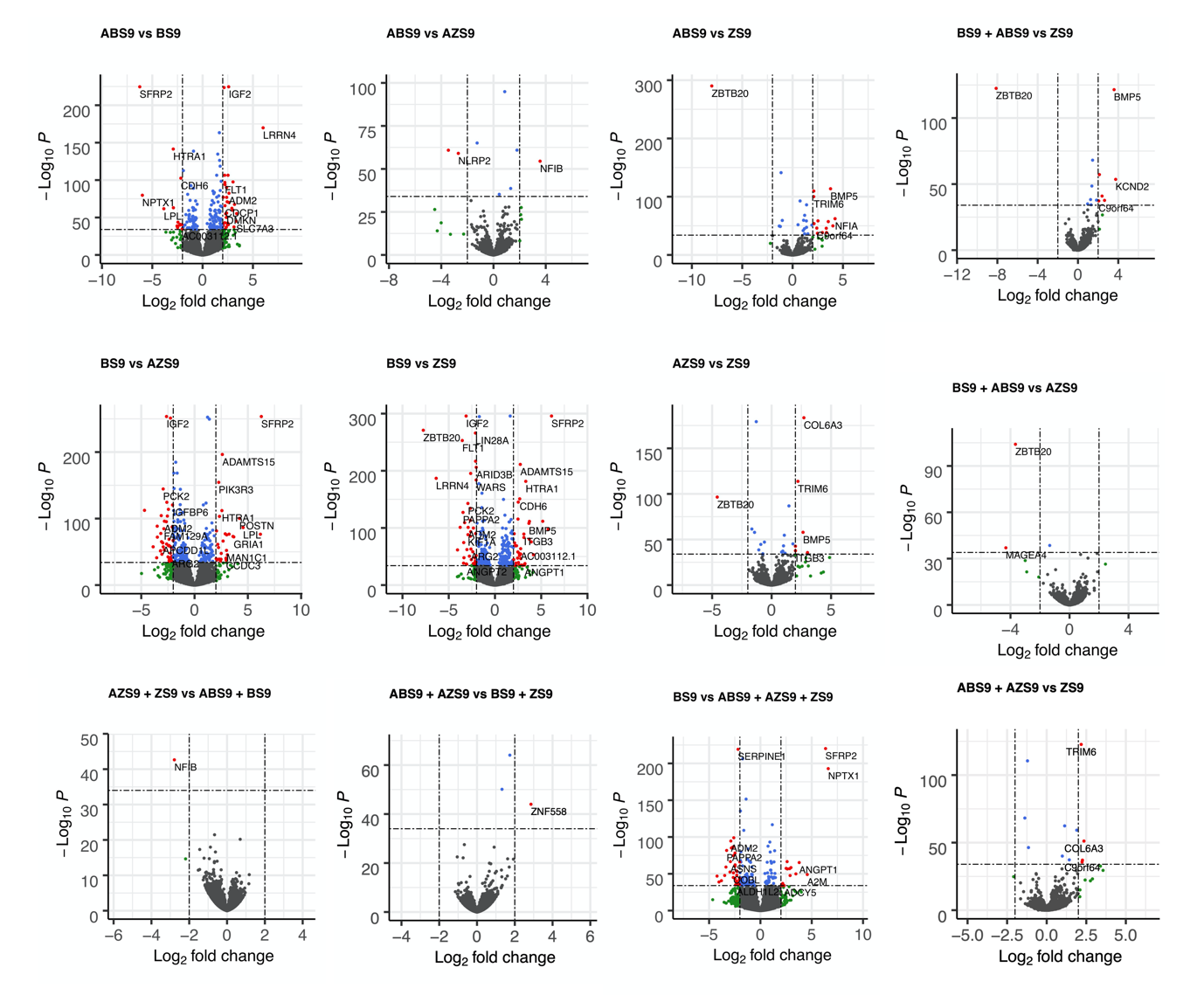


**Supplementary Figure S4.** **DEGs various volcano plot comparisons of the iAs from the 4 lines.**

Log_2_(fold change) versus log_10_(p-value) is shown for all genes with significantly expressed genes labelled in red. For labeling: green, unadjusted P≥10^e-35^ and absolute log_2_FC≥2; blue, unadjusted P≤10^e-35^ and absolute log_2_FC≤2; and red, unadjusted P≤10^e-35^ and absolute log_2_FC≥2.


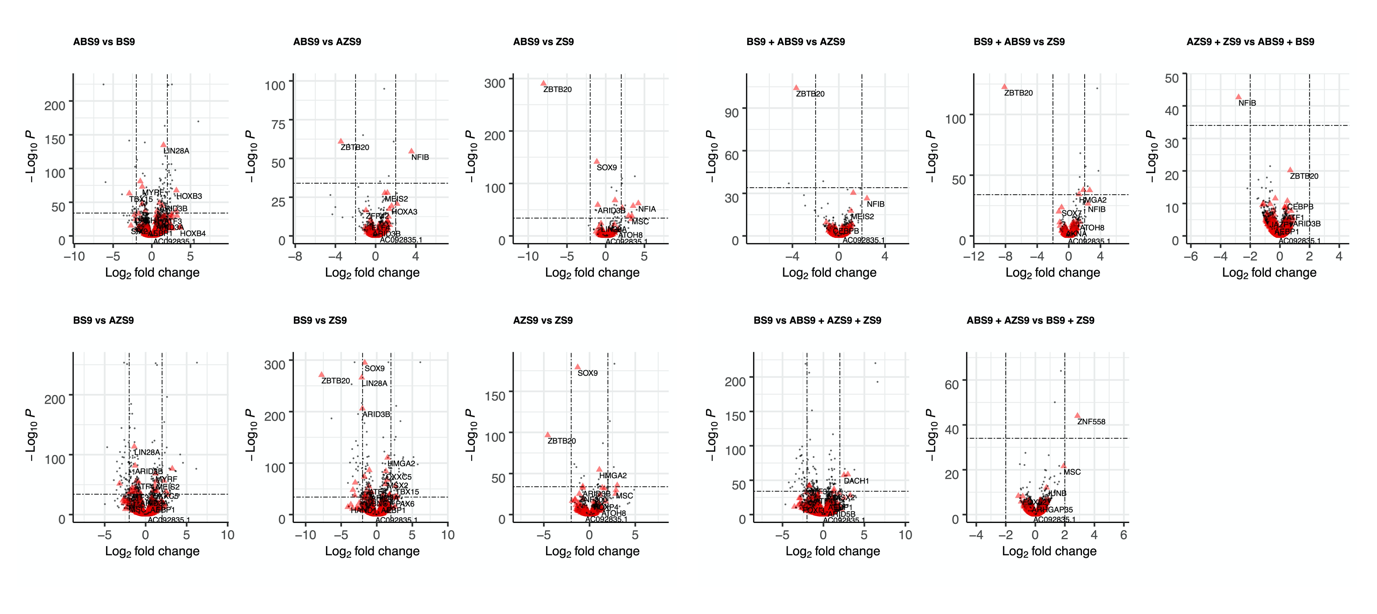


**Supplementary Figure S5.** **TFs various volcano plot comparisons of the iAs from the 4 lines.**

Log_2_(fold change) versus log_10_(p-value) is shown for all genes. Transcription factors are marked in red triangles. Cut-offs were set to unadjusted P≤10^e-35^ and absolute log_2_FC≥2


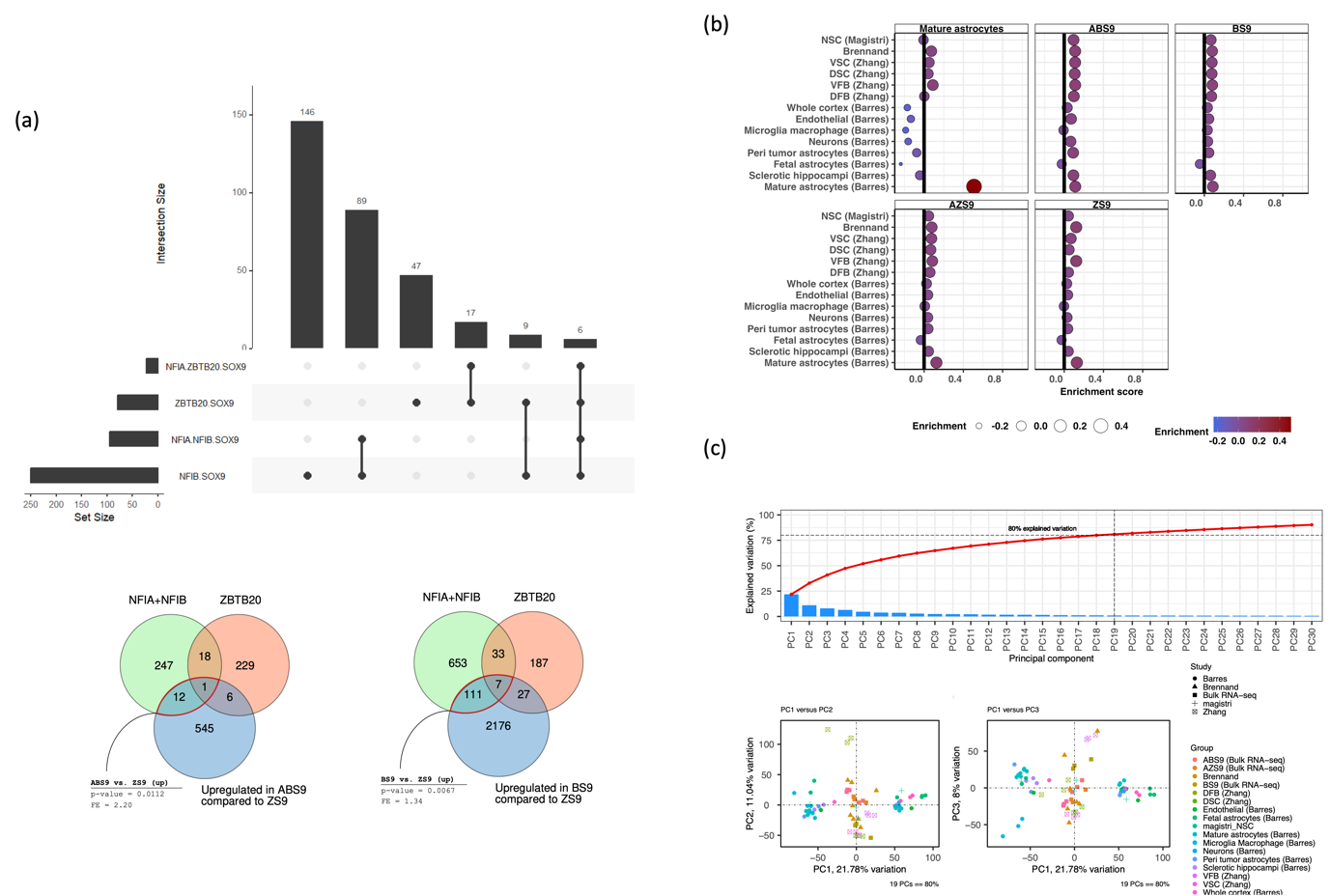


**Supplementary Figure S6. GRN analysis and comparison to published datasets of human astrocytes.**

(a) Upset plots comparing the 4 TF combinations to find unique and shared direct targets (top panel) and Venn diagrams showing overlap of putative TF binding sites based on their known motifs with TSS-proximal regions of open chromatin in astrocytes (bottom panel). Based on ATAC-seq dataset from (Donohue et al., 2022).

(b) Cell type enrichment represented by a points plot, based on several published datasets of astrocytes, neurons, and other glia (Bradley et al., 2019; Magistri et al., 2016; TCW et al., 2017; Zhang et al., 2016).

(c) Screen plot (top panel) and PCA pots (bottom panel) comparison between the 4 TF combinations and published datasets of astrocytes, neurons, and other glia.

**Supplementary Figure S7. A pairwise sample correlation plot.**

The 4 iAs lines (ABS9, AZS9, BS9 and ZS9) datasets were compared to published RNA-seq datasets of human astrocytes, neurons and other glial cells.

**Supplementary Experimental Procedures**

**RNA sequencing analysis**

Read count abundances were generated from raw data FASTQ files by pseudo-aligning these to the Homo sapiens GENCODE ‘comprehensive’ reference transcriptome (GRCh38.p12 / release 31) using Kallisto v0.46.0 (Bray et al., 2016), adjusting for GC content bias and bootstrapping 50x. Bootstrapped transcript isoform-level counts were then imported to R Programming Language v 3.6.1 (R) (Team, 2016) and summarized to gene level counts (adjusting for gene-length) using the tximport v1.13.12 package in R.

Protein coding genes (total = 19703) were then isolated from the raw counts based on GENCODE biotype keyword ‘protein_coding’. A gene with zero counts across all samples was removed (total = 1604). The raw counts of the remaining genes (total = 18099) (as a tximport object) were then converted to a DESeq2 v1.25.10 (Love et al., 2014) object for normalization with betaPrior set to FALSE. For downstream analyses, the negative binomial-distributed normalized counts were converted to regularized log (rlog) counts via the rlog function of DESeq2 in R, with blind set to FALSE. Variance-stabilized counts were also generated.

Differential expression analysis was conducted on the negative binomial-distributed normalized counts with FDR set at 5%. Following differential expression, log (base 2) fold changes (log_2_FC) were shrunk via the lfcshrink function of DESeq2. A gene was defined as differentially expressed if it passed Benjamini-Hochberg Q≤0.05 and absolute log_2_FC≥2. MA plots were generated using base R functions by plotting log_2_FC (y-axis) versus natural log normalized counts (x-axis). Prior to logging, normalized counts were shifted by +1 to avoid negative log values. Genes passing Benjamini-Hochberg Q≤0.05 and absolute log_2_FC≥2 were highlighted red. Volcano plots were generated using the EnhancedVolcano package v1.3.1 (Blighe, 2019). Plots were then plotted together using grid.arrange from the gridExtra v2.3 package. For labelling, cut-offs were set to unadjusted P≤10^e-35^ and absolute log_2_FC≥2. The top 50 most highly expressed genes were determined by first averaging the unlogged, normalized counts across each sample group and then ordering by decreasing order (high to low counts). To generate the heatmap, the heatmap.2 function of the gplots was used. Principal component analysis was performed using the PCAtools R package (Blighe et al., 2019). Regularized log counts were used as input. A bi-plot comparing PC1 and PC2 was generated, as was a pairs plot comparing PCs 1-5 on a pairwise basis. The main genes responsible for variation along each PC was generated via a loadings plot. Finally, correlation between PCs and metadata was generated via an eigencorplot. Supervised (filtered) clustering was performed using the Heatmap function of the ComplexHeatmap v2.1.0 package (Gu et al., 2016). Regularized log counts were converted to the Z scale (scaled by row/gene) and then clustered via 1 minus Pearson correlation distance and Ward's linkage (‘ward.D2’). A samples box-and-whisker plot of Z-scores was added to the heatmap bottom. A colour bar indicating the different sample groups was added at the heatmap top. To supervise clustering, genes from each differential expression analysis at Benjamini-Hochberg Q≤0.05 and absolute log_2_FC≥2 were included.

**Functional enrichment analysis**

Enrichment of differentially expressed genes was performed with enrichR v2.1. Only enriched Gene Ontology terms (GO-terms) / pathways passing enrichment p<0.05 were output. Heatmaps for GO-terms were generated using gplots package in R. Combined bar and line plots for pathway analysis were generated using Prism v. 8.4.2 (GraphPad) software.

**Comparison to public studies**

Three datasets were utilized:

1. Barres: GSE73721 (Zhang et al., 2016) - data downloaded as FPKM, oligodendrocyte samples removed, and then converted to z-scores via zFPKM (R).
2. Brennand: GSE97904 (TCW et al., 2017) - data downloaded as log2 CPM, Ensembl gene IDs converted to HGNC symbols via biomaRt (Durinck et al., 2005, 2009), and then converted to Z-scores
3. Zhang: GSE133489 (Bradley et al., 2019) - data downloaded as TPM and then converted to Z-scores

To reduce bias in downstream analyses, only common genes between each of these 3 datasets and our bulk RNA-seq samples were retained. Different methods were then employed for comparing these datasets.

The 4 datasets (3 public studies and our bulk RNA-seq) were merged and residual batch effects removed via limma’s removeBatchEffects function (Ritchie et al., 2015). PCA was then performed with PCAtools. Hierarchical clustering was performed using the dist and hclust functions in R, with Euclidean distance and Ward’s linkage (‘ward.D2’) respectively. A pairwise sample correlation plot was generated via corrplot v0.84.

Unbiased gene-set variation analysis (GSVA) using unfiltered data was performed using GSVA v1.33.1 (Hänzelmann et al., 2013) and using the C2 curated gene sets from Molecular Signatures Database (C2 MsigDB), with parameters min.sz=5, max.sz=999999, method=”ssgsea”, and abs.ranking=FALSE. Each of the 4 datasets was analyzed separately, with the input to GSVA in each case being Z-scores. Gene symbols were converted to Entrez IDs via biomaRt in order to match to the C2 gene sets. Gene symbols that did not match were removed. Separate GSVA enrichments were also made against just KEGG pathways contained within the C2 gene sets.

The top 100 most activated signatures / pathways in each sample sub-group (e.g., mature astrocytes, fetal astrocytes, etc.) were then compared to the top 100 in each of our bulk RNA-seq groups, with this comparison then illustrated via Venn diagrams.

For each of the 3 public studies and their sample sub-groups (e.g., fetal astrocytes, mature astrocytes, etc), the top 100 most highly expressed genes (based on Z-scores) was determined and then saved as a gene set object for GSVA. Our bulk RNA-seq samples were then enriched against these gene sets in order to gauge the level of enrichment of the public studies’ signatures in our data.

The resulting enrichment scores were then averaged across sample sub-group and plot as a single value (per sample sub-group) via corrplot v0.84 (left panel in PublicStudies/CelltypeEnrichment.pdf). These were also plot as a points plot via ggplot2 (Wickham, 2009) (right panel in PublicStudies/CelltypeEnrichment.pdf).

Barres FPKM data was transformed to Z-scale via zFPKM (R / Bioconductor) and then row (gene) standardised, while Zhang TPM data was standardised as is. These datasets were merged with our study data based on common gene symbols, and then any potential study-specific batch effects removed via limma::removeBatchEffect(). A 'signature' for each Barres and Zhang cell-type was then defined based on the top 100 expressed genes based on mean standardised score. GSVA (R / Bioconductor) was then used to gauge the level of enrichment of each signature in our study data. To serve as a positive control, we enriched the Barres Mature Astrocytes against its own 100 gene signature.

**Cell type analysis** for DEGs from both primary and induced astrocytes from *in vivo* RNA-seq data, was performed using Enrichr (Chen et al., 2013; Kuleshov et al., 2016; Xie et al., 2021).

**GRN analysis.** Human astrocyte ATAC-Seq data were downloaded from GEO (dataset 1: GSM5680687, dataset 2: GSE113480) (Donohue et al., 2022; Song et al., 2019). Peak annotation was performed with the genecode human reference genome v19 using the ChIPseeker and ChIPpeakAnno R packages (TSS region -3000 +3000). Peaks were transferred to Homer, in which motif enrichment was carried out using the annotatePeaks function. Corresponding targets were extracted and plotted as upset plot using R package UpSetR. DEGs (adj pV≤0.05, |log2FC|≥0.3) from the experimental RNA-Seq data were extracted and compared with the target genes of the TFs derived from ATAC-Seq and plotted as venn diagram using the VennDiagram package. Motif and target occurence were analyzed using chi square test (pV<0.05) and fold enrichment. Analyses were performed in R (version 4.2.1).

Note that this approach will identify likely direct targets binding in the promoter-proximal region but is unable to detect long-range enhancer mediated binding, as confident assignment of enhancer sites to promoters without additional experiments remains challenging. For each combination of TFs that was used in this study we then overlapped their putative direct targets with upregulated genes in one combination with respect to the other, repeating the procedure for the downregulated genes. Using a Chi-squared test with Yates’ continuity correction we tested if more of the putative direct targets were found among the differentially expressed genes than expected by chance.

Following either a Bonferroni or a less conservative BH FDR correction at 0.05 for these tests to find overlaps.

**Analysis of in vivo RNA-seq data.** Mixed reads were segregated into the Rat and human reads using SARGASSO (Qiu et al., 2018). Rat and Human Reads were aligned to the Rattus_norvegicus.mRatBN7.2and Grch38 reference genome, respectively, using the STAR aligner (Dobin et al., 2013). Count files generated from aligned BAM files using htseq-count (Putri et al., 2022). All samples were merged into a single merged file for downstream analysis. Merged counts files were further analyzed using Deseq2 (Love et al., 2014). Count Normalization was performed using the VST method of DEseq2, and then normalized reads were plotted using R.

**Supplementary Table S1. List of primers for genotyping PCR**

| **Locus** | **PCR type** | **Primer binding site** | **Primer Sequence** |
| --- | --- | --- | --- |
| hROSA26 | Locus PCR | Genome (5’) | GAGAAGAGGCTGTGCTTCGG |
|  |  | Genome (3’) | ACAGTACAAGCCAGTAATGGAG |
|  | 5’INT PCR | Genome (5’) | GAGAAGAGGCTGTGCTTCGG |
|  |  | Splice Acceptor | AAGACCGCGAAGAGTTTGTCC |
|  | 3’INT PCR | rtTA | GAAACTCGCTCAAAAGCTGGG |
|  |  | Genome (3’) | ACAGTACAAGCCAGTAATGGAG |
|  | 3’BB PCR | rtTA | GAAACTCGCTCAAAAGCTGGG |
|  |  | Vector Backbone (3’) | TGACCATGATTACGCCAAGC |
| AAVS1 | Locus PCR | Genome (5’) | CTGTTTCCCCTTCCCAGGCAG |
|  |  | Genome (3’) | TGCAGGGGAACGGGGCTCA |
|  | 5’INT PCR | Genome (5’) | CTGTTTCCCCTTCCCAGGCAG |
|  |  | Puromycin | TCGTCGCGGGTGGCGAG |
|  | 3’INT PCR | Transgene | transgene specific sequence |
|  |  | Genome (3’) | TGCAGGGGAACGGGGCTCA |
|  | 3’BB PCR | Transgene | transgene specific sequence |
|  |  | Vector Backbone (3’) | ATGCTTCCGGCTCGTATGTT |

**Supplementary Table S2. List of primary antibodies used in this study**

| **1^st^ antibodies** | **Supplier** | **Host** |
| --- | --- | --- |
| β3-tubulin | Proteintech | Mouse |
| Cx43 | Abcam | Rabbit |
| GFAP | GeneTex | Chicken |
| Human Anti-Nuclei | Millipore | Mouse |
| Human GFAP | TakaraBio | Mouse |
| Nanog | R&D | Goat |
| OCT4 | Cell Signaling Technology | Mouse |
| PAX6 | abcam | Rabbit |
| S100β | GeneTex | Rabbit |
| Synapsin-1 | Abcam | Rabbit |
| Vimentin | Millipore | Mouse |

**Supplementary Table S3. List of primers used for qPCR analysis**

| **Gene** | **Forward Sequence** | **Reverse Sequence** |
| --- | --- | --- |
| *ALDH1L1* | AGCAGTGACGGGTGAT | GTGATTGGACAGAGCC |
| *AQP4* | GGCCGTAATCTGACTCCCAG | TGTGGGTCTGTCACTCATGC |
| *CD44* | AGTTTTGGTGGCACGCAG | GCCTCCGTCCGAGAGATG |
| *CXCL10* | GGAACCTCCAGTCTCAGCACC | AGGTACAGCGTACGGTTCTAG |
| *GFAP* | GTCCCCCACCTAGTTTGCAG | TAGTCGTTGGCTTCGTGCTT |
| *IL-6* | ATGAACTCCTTCTCCACAAGC | AAGAGGTGAGTGGCTGTCT |
| *NFIA* | GCCATCTCCAACCACACTGA | GGCTGCTGGATAATGGGTGA |
| *NFIA endo* | GGTTTTTCTCCCCTTCCGCT | CCACCTGACCCCTTCTCAAC |
| *NFIB* | ACATCCACTGAAGCCTACACA | CAGGTACCAGGACTGTTGCT |
| *NFIB endo* | ACAGTAGGCAGAACAGTCGC | TGCAGTGTAGCTGTGAAGCA |
| *PBGD1* | ATTACCCCGGGAGACTGAAC | GGCTGTTGCTTGGACTTCTC |
| *S100β* | TGTAGACCCTAACCCGGAGG | TGCATGGATGAGGAACGCAT |
| *SOX9* | AGACTTCTGAACGAGAGCGAG | GTTCTTCACCGACTTCCTCCG |
| *SOX9 endo* | CTGCTCGTCGGTCATCTTCA | CCTTCCTAAGTGCTCGCCG |
| *VIM* | TGGACCAGCTAACCAACGAC | GCCAGAGACGCATTGTCAAC |
| *ZBTB20* | AGCAGGAGATGGAGGACGAT | TCCTCGGATTCGTTGCGT |
| *ZBTB20 endo* | GAGTCAAAGAGGAGGTGGGC | AAGGAGGGAAATGGCTGTGG |

**Supplementary References**

Blighe, K. (2019). Publication-ready volcano plots with enhanced colouring and labeling. R-Package.

Blighe, K., Lewis, M., and Lun, A. (2019). PCAtools: Everything Principal Components Analysis.

Bradley, R.A., Shireman, J., McFalls, C., Choi, J., Canfield, S.G., Dong, Y., Liu, K., Lisota, B., Jones, J.R., Petersen, A., et al. (2019). Regionally specified human pluripotent stem cell-derived astrocytes exhibit different molecular signatures and functional properties. Development *146*, dev.170910.

Bray, N.L., Pimentel, H., Melsted, P., and Pachter, L. (2016). Near-optimal probabilistic RNA-seq quantification. Nat. Biotechnol. *34*, 525–527.

Chen, E.Y., Tan, C.M., Kou, Y., Duan, Q., Wang, Z., Meirelles, G. V., Clark, N.R., and Ma’ayan, A. (2013). Enrichr: interactive and collaborative HTML5 gene list enrichment analysis tool. BMC Bioinformatics *14*.

Dobin, A., Davis, C.A., Schlesinger, F., Drenkow, J., Zaleski, C., Jha, S., Batut, P., Chaisson, M., and Gingeras, T.R. (2013). STAR: ultrafast universal RNA-seq aligner. Bioinformatics *29*, 15–21.

Donohue, L.K.H., Guo, M.G., Zhao, Y., Jung, N., Bussat, R.T., Kim, D.S., Neela, P.H., Kellman, L.N., Garcia, O.S., Meyers, R.M., et al. (2022). A cis-regulatory lexicon of DNA motif combinations mediating cell-type-specific gene regulation. Cell Genomics *2*, 100191.

Durinck, S., Moreau, Y., Kasprzyk, A., Davis, S., De Moor, B., Brazma, A., and Huber, W. (2005). BioMart and Bioconductor: A powerful link between biological databases and microarray data analysis. Bioinformatics *21*, 3439–3440.

Durinck, S., Spellman, P.T., Birney, E., and Huber, W. (2009). Mapping identifiers for the integration of genomic datasets with the R/ Bioconductor package biomaRt. Nat. Protoc. *4*, 1184–1191.

Gu, Z., Eils, R., and Schlesner, M. (2016). Complex heatmaps reveal patterns and correlations in multidimensional genomic data. Bioinformatics *32*, 2847–2849.

Hänzelmann, S., Castelo, R., and Guinney, J. (2013). GSVA: gene set variation analysis for microarray and RNA-Seq data. BMC Bioinformatics *14*, 7.

Kuleshov, M. V., Jones, M.R., Rouillard, A.D., Fernandez, N.F., Duan, Q., Wang, Z., Koplev, S., Jenkins, S.L., Jagodnik, K.M., Lachmann, A., et al. (2016). Enrichr: a comprehensive gene set enrichment analysis web server 2016 update. Nucleic Acids Res. *44*, W90–W97.

Love, M.I., Huber, W., and Anders, S. (2014). Moderated estimation of fold change and dispersion for RNA-seq data with DESeq2. Genome Biol. *15*.

Magistri, M., Khoury, N., Mazza, E.M.C., Velmeshev, D., Lee, J.K., Bicciato, S., Tsoulfas, P., and Faghihi, M.A. (2016). A comparative transcriptomic analysis of astrocytes differentiation from human neural progenitor cells. Eur. J. Neurosci. *44*, 2858–2870.

Putri, G.H., Anders, S., Pyl, P.T., Pimanda, J.E., and Zanini, F. (2022). Analysing high-throughput sequencing data in Python with HTSeq 2.0. Bioinformatics *38*, 2943–2945.

Qiu, J., Dando, O., Baxter, P.S., Hasel, P., Heron, S., Simpson, T.I., and Hardingham, G.E. (2018). Mixed-species RNA-seq for elucidation of non-cell-autonomous control of gene transcription. Nat. Protoc. 2018 1310 *13*, 2176–2199.

Ritchie, M.E., Phipson, B., Wu, D., Hu, Y., Law, C.W., Shi, W., and Smyth, G.K. (2015). Limma powers differential expression analyses for RNA-sequencing and microarray studies. Nucleic Acids Res. *43*, e47.

Song, M., Yang, X., Ren, X., Maliskova, L., Li, B., Jones, I.R., Wang, C., Jacob, F., Wu, K., Traglia, M., et al. (2019). Mapping cis-regulatory chromatin contacts in neural cells links neuropsychiatric disorder risk variants to target genes. Nat. Genet. 2019 518 *51*, 1252–1262.

TCW, J., Wang, M., Pimenova, A.A., Bowles, K.R., Hartley, B.J., Lacin, E., Machlovi, S.I., Abdelaal, R., Karch, C.M., Phatnani, H., et al. (2017). An Efficient Platform for Astrocyte Differentiation from Human Induced Pluripotent Stem Cells. Stem Cell Reports *9*, 600–614.

Team, R.C. (2016). R: A Language and Environment for Statistical Computing. R Found. Stat. Comput.

Wickham, H. (2009). ggplot2 (New York, NY: Springer New York).

Xie, Z., Bailey, A., Kuleshov, M. V., Clarke, D.J.B., Evangelista, J.E., Jenkins, S.L., Lachmann, A., Wojciechowicz, M.L., Kropiwnicki, E., Jagodnik, K.M., et al. (2021). Gene Set Knowledge Discovery with Enrichr. Curr. Protoc. *1*, e90.

Zhang, Y., Sloan, S.A., Clarke, L.E., Caneda, C., Plaza, C.A., Blumenthal, P.D., Vogel, H., Steinberg, G.K., Edwards, M.S.B., Li, G., et al. (2016). Purification and Characterization of Progenitor and Mature Human Astrocytes Reveals Transcriptional and Functional Differences with Mouse. Neuron *89*, 37–53.
